# Host-derived reactive oxygen species trigger activation of the *Candida albicans* transcription regulator Rtg1/3

**DOI:** 10.1371/journal.ppat.1011692

**Published:** 2023-09-28

**Authors:** Mazen Oneissi, Melissa R. Cruz, Bernardo Ramírez-Zavala, Elena Lindemann-Perez, Joachim Morschhäuser, Danielle A. Garsin, J. Christian Perez

**Affiliations:** 1 Department of Microbiology and Molecular Genetics, McGovern Medical School, The University of Texas Health Science Center at Houston, Houston, United States of America; 2 Institute of Molecular Infection Biology, University of Würzburg, Würzburg, Germany; University of Georgia, UNITED STATES

## Abstract

The signals that denote mammalian host environments and dictate the activation of signaling pathways in human-associated microorganisms are often unknown. The transcription regulator Rtg1/3 in the human fungal pathogen *Candida albicans* is a crucial determinant of host colonization and pathogenicity. Rtg1/3’s activity is controlled, in part, by shuttling the regulator between the cytoplasm and nucleus of the fungus. The host signal(s) that Rtg1/3 respond(s) to, however, have remained unclear. Here we report that neutrophil-derived reactive oxygen species (ROS) direct the subcellular localization of this *C*. *albicans* transcription regulator. Upon engulfment of *Candida* cells by human or mouse neutrophils, the regulator shuttles to the fungal nucleus. Using genetic and chemical approaches to disrupt the neutrophils’ oxidative burst, we establish that the oxidants produced by the NOX2 complex–but not the oxidants generated by myeloperoxidase–trigger Rtg1/3’s migration to the nucleus. Furthermore, screening a collection of *C*. *albicans* kinase deletion mutants, we implicate the *MKC1* signaling pathway in the ROS-dependent regulation of Rtg1/3 in this fungus. Finally, we show that Rtg1/3 contributes to *C*. *albicans* virulence in the nematode *Caenorhabditis elegans* in an ROS-dependent manner as the *rtg1* and *rtg3* mutants display virulence defects in wild-type but not in ROS deficient worms. Our findings establish NOX2-derived ROS as a key signal that directs the activity of the pleiotropic fungal regulator Rtg1/3.

## Author summary

*Candida albicans* is a ubiquitous fungus of humans. It inhabits various compartments of the human body including oral cavity and gastrointestinal tract. *C*. *albicans* is also a leading cause of life-threatening disseminated infections. As with other human-associated microorganisms, the exact nature of the signals sensed by this fungus inside host tissues is often unknown. We have established that the *C*. *albicans* regulatory system Rtg1/3 is pivotal for the fungus to colonize and proliferate inside the mammalian host, implying that the regulator’s activity may respond to one or more cues that denote the host environment. In this study, we sought to pinpoint the cue(s) and signaling molecules that direct the regulator’s activity inside the host. We report that host-derived reactive oxygen species determine the activation of this regulator by promoting its translocation from the cytoplasm to the nucleus of the fungus. As a signal that dictates the activity of Rtg1/3, reactive oxygen species may concomitantly direct the activation of additional pathways required for *Candida* to endure in the host because Rtg1/3 constitutes a central node of a rather large regulatory network driving mammalian host colonization.

## Introduction

Host-associated microbes harbor cellular pathways that must be turned on or off in response to cues in the host environment. Indeed, nutrients, pH, ions, and other host-produced molecules have been shown to impact microbial gene expression profiles. However, the exact nature of the signals sensed by microorganisms inside host tissues is often unknown. The fungus *Candida albicans* inhabits the digestive tract of most healthy humans and is the most common cause of life-threatening, invasive fungal infections [1–3]. While progress has been made in outlining fungal and host factors that contribute to invasive candidiasis [4–6], the cues in the host environment that *C*. *albicans* gauge and that elicit the activation or deactivation of signaling pathways in the fungal cell are still poorly defined [7, 8].

The *Candida albicans* heterodimeric transcription regulator Rtg1/3 is a key determinant of host colonization and pathogenicity. We have established that the *C*. *albicans rtg1* and *rtg3* mutants display impaired murine gut colonization and reduced virulence in the mouse tail-vein infection model [9]. Rtg1/3 regulates the expression of an eclectic set of transcripts in *C*. *albicans*. For example, the regulator has been shown to control transcription of galactose utilization genes [10] and of several genes involved in the synthesis of sphingolipids’ building blocks [11]. By contrast, in the free-living model yeast *Saccharomyces cerevisiae*, Rtg1/3 has primarily been investigated in the context of the cellular response to the absence of mitochondrial DNA [12–14] and nitrogen sources [15, 16]. In both *C*. *albicans* and *S*. *cerevisiae* the activity of the regulator is controlled, in part, by shuttling the proteins between cytoplasm and nucleus [11, 15, 16]. What signal(s) and/or signaling pathway(s) lie upstream of Rtg1/3, particularly in *C*. *albicans*, remains underexplored.

Neutrophils, also termed polymorphonuclear leukocytes (or PMNs), are the most abundant phagocyte population in humans and are critical for *C*. *albicans* clearance [17–19]. These cells are usually the earliest to be recruited to sites of infection and are endowed with powerful oxidative and non-oxidative killing mechanisms [20]. Upon stimulation of pattern recognition receptors, downstream kinase activation and Ca^2+^-mediated signaling trigger neutrophils to assemble a large protein complex known as nicotinamide adenine dinucleotide phosphate (NADPH) oxidase (NOX2). The assembly results in a functional enzymatic multimeric protein that reduces molecular oxygen to superoxide anion. The neutrophil enzymes superoxide dismutase and myeloperoxidase (MPO) further convert these highly reactive radicals to hydrogen peroxide and hypochlorous acid, respectively. Collectively known as reactive oxygen species (ROS), the mix of these intermediates act both as efficient antimicrobials and as short-lived signaling molecules [17, 20]. Oxidative radical-forming mechanisms appear to damage fungi by producing protein modifications, nucleic acid breaks and lipid peroxidation [21].

Here we investigate the host signal(s) that *C*. *albicans* Rtg1/3 may respond to. The observation that the regulator translocated to the nucleus of the fungus upon *Candida* engulfment by human and mouse neutrophils prompted us to examine the involvement of ROS. Using a combination of chemical and genetic approaches to disrupt the PMN’s oxidative burst pathway at various steps, we establish that ROS generated by NOX2 elicit the translocation of the regulator to the fungal nucleus. Consistent with the notion that ROS are a cue that dictates Rtg1/3 activation, we show that, in the nematode *Caenorhabditis elegans*, the fungus relies on this regulator to withstand the worm’s ROS defense system. To our knowledge, this is the first fungal transcription regulator that has been shown to translocate to the nucleus of the fungal cell upon infection of a mammalian host.

## Results

### Rtg1/3 translocate to the fungal nucleus upon engulfment of *Candida* by neutrophils

The heterodimeric transcription regulator Rtg1/3 is required for *C*. *albicans* to colonize multiple murine organs [9, 22], implying that the regulator’s activity in this fungal species may respond to one or more cues that denote the host environment. We have established that a key point of control of the Rtg1/3 proteins is the migration from cytoplasm to nucleus [11]. Under laboratory culture conditions, nutrient deprivation or inhibition of sphingolipid synthesis cause Rtg1p and Rtg3p to accumulate in the nucleus of *Candida* [11]. In the context of the host environment, on the other hand, we observed that the Rtg3 protein shuttled to the *Candida* nucleus upon engulfment of the fungus by human neutrophils [11]. Consistent with this observation, deletion of the regulator rendered the fungus more susceptible to killing by these phagocytes [11].

**Fig 1:**
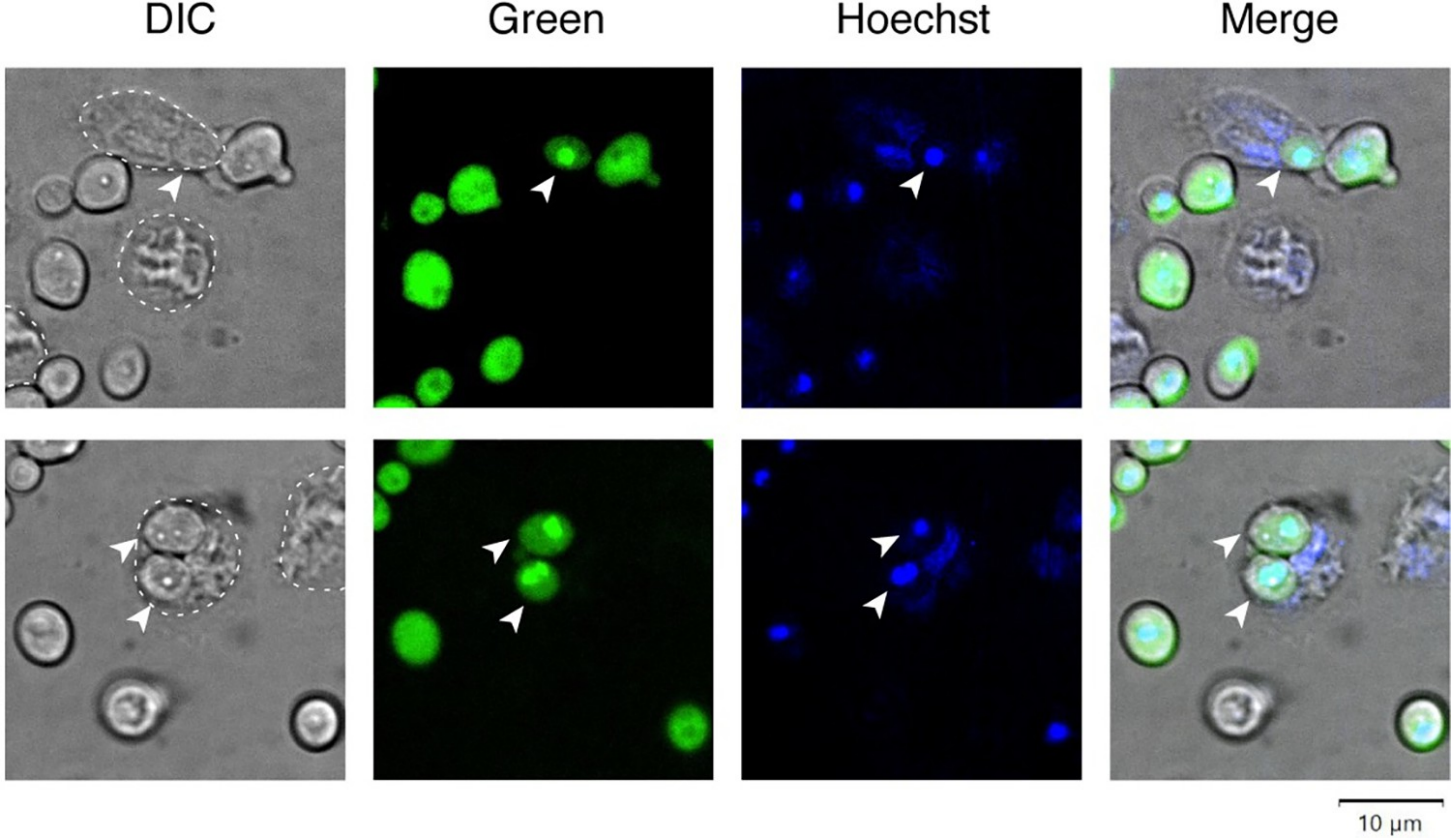
The *C*. *albicans* transcription regulator Rtg1/3 translocates to the fungal nucleus upon phagocytosis by neutrophils. *C*. *albicans* expressing the reporter GFP-Rtg3 was incubated with bone marrow derived neutrophils isolated from wild-type C57BL/6J mice. Before mixing with neutrophils, the *Candida* cells were pre-incubated with Hoechst to stain the fungal nucleus. Notice that the reporter is distributed throughout the fungal cells in non-phagocytosed *Candida*. Upon uptake by neutrophils, the reporter accumulates in the *Candida* nucleus (arrowheads). Shown are two representative images taken 15 minutes after addition of fungal cells to neutrophils. The edges of the neutrophils are outlined in the DIC images.

**Fig 2:**
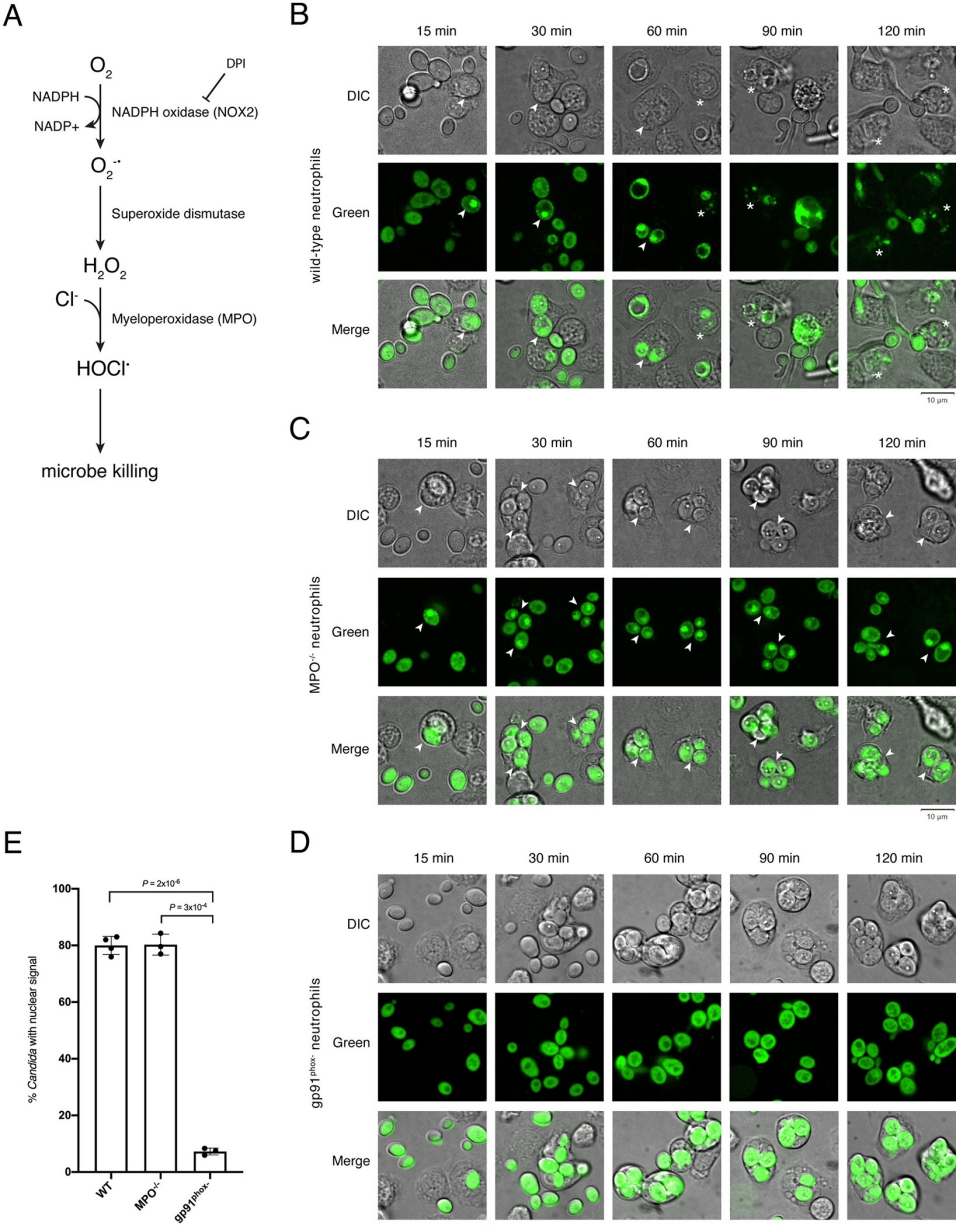
Neutrophil’s oxidative burst is required for Rtg1/3 migration to the *Candida* nucleus. (**A**) Schematic of the neutrophil oxidative burst pathway. NADPH, nicotinamide adenine dinucleotide phosphate; DPI, diphenyleneiodonium; H_2_O_2_, hydrogen peroxide; HOCl^-^, hypochlorous acid. (**B**, **C** and **D**) Subcellular localization of the GFP-Rtg3 reporter in *Candida* cells upon uptake by wild-type (**B**), MPO^-/-^ (**C**), or gp91^phox^ KO (**D**) neutrophils. Notice that phagocytosed *Candida* cells remain intact inside MPO^-/-^ and gp91^phox^ KO neutrophils throughout the duration of the experiment whereas fungal cells phagocytosed by wild-type PMNs appear fragmented and decaying (marked with asterisks) at later time points (starting at 60 min). The fluorescent reporter accumulates in the fungal nucleus (arrowheads) in *Candida* cells phagocytosed by MPO^-/-^ neutrophils but not in those taken up by gp91^phox^ KO PMNs. (**E**) Quantification of phagocytosed *C*. *albicans* cells displaying accumulation of the reporter in the fungal nucleus. A minimum of 100 *Candida* cells were scored per experiment per condition. At least three independent experiments were performed. Plotted are the means ± SD. Statistical analysis was conducted using Student’s *t*-test (two-tailed, two-sample unequal variance).

To determine whether the regulator’s nuclear translocation also occurred in mouse PMNs, we incubated murine bone marrow derived neutrophils with a *C*. *albicans* strain expressing GFP-Rtg3p. The fluorescent signal was largely cytoplasmic in *Candida* cells that remained in the medium, unphagocytosed (Figs 1 and S1). Upon engulfment by mouse neutrophils, however, the fluorescent signal rapidly accumulated in a single spot inside the *Candida* cell which corresponded to the fungal nucleus as determined by DNA staining with Hoechst. At later time points, the fluorescent signal from the reporter dissipated (Fig 2) as the phagocytosed *Candida* succumbed to killing by wild-type neutrophils, as expected.

As a first step to illuminate the host environment(s) that Rtg1/3 responds to, we sought to dissect the neutrophil features that lead to the regulator’s nuclear localization. Because experimenting with human neutrophils requires human blood donors and this may introduce genetic differences as confounding factor, we chose mouse PMNs for all the experiments described in this report. However, we note that the *C*. *albicans* strain expressing GFP-Rtg3p displays essentially the same behavior in both mouse PMNs and freshly isolated human neutrophils (S1 Fig).

### Rtg1/3 localize to the fungal nucleus in myeloperoxidase-deficient neutrophils

The hallmark of neutrophils is the oxidative burst which is known to generate oxidants (reactive oxygen species, ROS) that react with and damage microbial macromolecules, ultimately leading to the killing of microbes. The oxidative burst in mammals consists of sequential steps (reviewed in [20]), some of which can be inhibited (Fig 2A). In the first step, O_2_ is converted to oxygen radicals (O_2_^-•^) by the enzyme NADPH oxidase (NOX2). Oxygen radicals are then converted to hydrogen peroxide (H_2_O_2_), a step catalyzed by the enzyme superoxide dismutase. Myeloperoxidase (MPO) is the last enzyme in the cascade and utilizes chloride anions to convert H_2_O_2_ into hypochlorous acid (HOCl^•^) which is the main effector in the killing of microbes. We hypothesized that one or several of these ROS species may trigger Rtg1/3 translocation to the nucleus. To test this hypothesis, we sought to evaluate whether disrupting specific steps of the oxidative burst cascade in neutrophils, either genetically or chemically, would impair Rtg1/3 nuclear localization.

We first employed neutrophils derived from MPO^-/-^ mice (B6.129X1-*Mpo*^*tm1Lus*^/J). While hypochlorous acid (HOCl^•^) is undetectable in leukocytes derived from these mice, superoxide (O_2_^-•^) production remains comparable between MPO^-/-^ and wild-type animals and cells [23]. We incubated MPO^-/-^ mouse bone marrow derived neutrophils with the *C*. *albicans* strain expressing GFP-Rtg3p. As expected, and consistent with a previous report [23], the MPO^-/-^ neutrophils were unable to kill many of the engulfed *Candida* cells within the time frame of our experiment (Fig 2C). In contrast to wild-type mouse neutrophils (Fig 2B), in which the majority of engulfed *Candida* cells appeared amorphous or disintegrated by 60–90 minutes post inoculation, the *Candida* cells phagocytosed by the MPO^-/-^ neutrophils remained intact even after 120 minutes post inoculation (Fig 2C). In fact, there were multiple *C*. *albicans* cells inside each MPO^-/-^ neutrophil at the later time point. Of importance to this report, the fluorescent reporter still accumulated in the nucleus of *Candida* cells phagocytosed by MPO^-/-^ neutrophils, at every time point evaluated (Fig 2C). From this result we conclude that hypochlorous acid (HOCl^•^) in the host environment does not influence the subcellular localization of the Rtg1/3 regulator. Furthermore, this finding rules out the possibility that the shuttling of the regulator to the nucleus simply reflects a generic effect from dying fungal cells.

### ROS produced by the NOX2 complex trigger Rtg1/3’s migration to the nucleus

We next used neutrophils derived from gp91^phox^ KO mice (B6.129S-*Cybb*^*tm1Din*^/J). gp91^phox^ is the catalytic subunit of the NADPH oxidase (NOX2) [20]; consequently, these KO mice are deficient in phagocyte superoxide production. As shown in Fig 2D and 2E, and in contrast to the observations with the MPO^-/-^ PMNs, the number of engulfed *Candida* cells displaying accumulation of fluorescent signal in the nucleus was significantly reduced in gp91^phox^ KO neutrophils.

**Fig 3:**
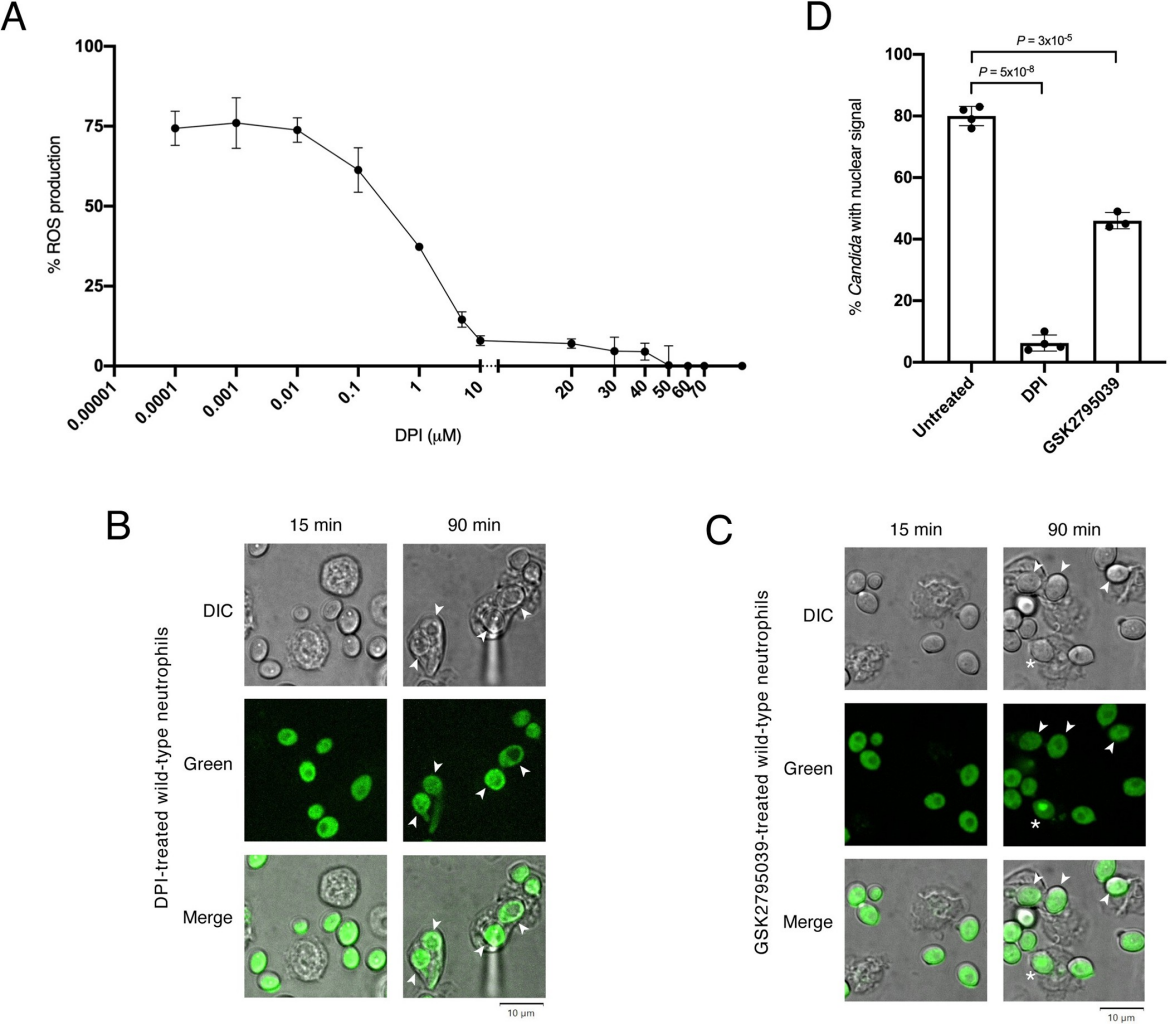
NOX2 activity in neutrophils in necessary for Rtg1/3 migration to the *Candida* nucleus. (**A**) DPI-ROS dose-response curve. Wild-type C57BL/6J mouse neutrophils were isolated from femurs and incubated in 96-well plates with various DPI concentrations. Percentage of ROS production was calculated relative to controls that did not contain DPI. Each DPI concentration was evaluated in triplicates. Plotted are the means ± S.D. (**B** and **C**) Subcellular localization of the GFP-Rtg3 reporter in *Candida* cells upon uptake by wild-type neutrophils treated with either DPI (**B**) or GSK2795039 [30 μM] (**C**). Arrowheads point to phagocytosed fungal cells displaying reporter in the *Candida* cytoplasm. Asterisk indicates reporter accumulation in the nucleus. (**D**) Quantification of phagocytosed *C*. *albicans* cells displaying accumulation of the reporter in the fungal nucleus. A minimum of 100 *Candida* cells were scored per experiment per condition. At least three independent experiments were performed. Plotted are the means ± SD. Statistical analysis was conducted using Student’s *t*-test (two-tailed, two-sample unequal variance).

To independently evaluate the involvement of NOX2, we used an inhibitor of this enzyme in neutrophils, diphenyleneiodonium chloride (DPI). DPI has been shown to reduce intracellular ROS production selectively and irreversibly in PMA-activated human neutrophils in a concentration dependent manner [24]. However, to our knowledge, there are no reports of how DPI may impact mouse neutrophils. Therefore, we first probed whether DPI has any effect on ROS production in mouse phagocytes. We measured ROS production in wild-type mouse neutrophils treated with various DPI concentrations and found that the drug reduced ROS levels in a concentration dependent manner (Fig 3A). Under our experimental conditions, DPI concentrations >10μM resulted in >95% ROS reduction (Fig 3A). We chose to use 30μM DPI to inhibit NOX2 in the following assays as this is the concentration that has been used in experiments with human neutrophils [24]. (Doses up to 40μM DPI have been shown to be noncytotoxic and have no general adverse effects on cell function.)

DPI-treated mouse neutrophils were able to phagocytose *C*. *albicans* at levels comparable to untreated controls (compare Figs 3B and 2B). The yeast cells, however, remained intact inside the DPI-treated neutrophils, which is consistent with the notion that impairing the oxidative burst renders the neutrophils unable to kill *Candida*. To evaluate the effects of targeting NOX2 on the subcellular localization of Rtg1/3 in engulfed *Candida*, we removed the drug from the medium before adding the fungal cells. This washing step was necessary to avoid any direct effect of DPI on *C*. *albicans* itself. As shown in Fig 3B and 3D, and in agreement with the observations with the gp91^phox^ KO neutrophils, the number of engulfed *Candida* cells displaying accumulation of fluorescent signal in the nucleus was significantly reduced in DPI-treated neutrophils compared to untreated controls.

The addition of the small molecule GSK2795039, another inhibitor of NOX2 [25], also reduced the nuclear localization of the reporter (Fig 3C and 3D), albeit its effect was not as prominent as DPI. The differences in the effects produced by these two drugs may be due to the fact that, in our assays, we washed away the drugs (DPI or GSK2795039) before adding *C*. *albicans*. This step minimizes any effect resulting from the drug targeting the fungus rather than the PMNs. DPI is probably better suited to this experimental setup because it targets NOX2 irreversibly whereas GSK2795039 does so reversibly [25]. Taken together, the genetic and chemical approaches used here demonstrate that the regulator’s subcellular localization in *Candida* does respond to the neutrophil’s oxidative burst and is triggered by ROS generated downstream of NOX2 but upstream of MPO.

### H_2_O_2_ is sufficient to promote Rtg1/3’s migration to the nucleus *in vitro*

**Fig 4:**
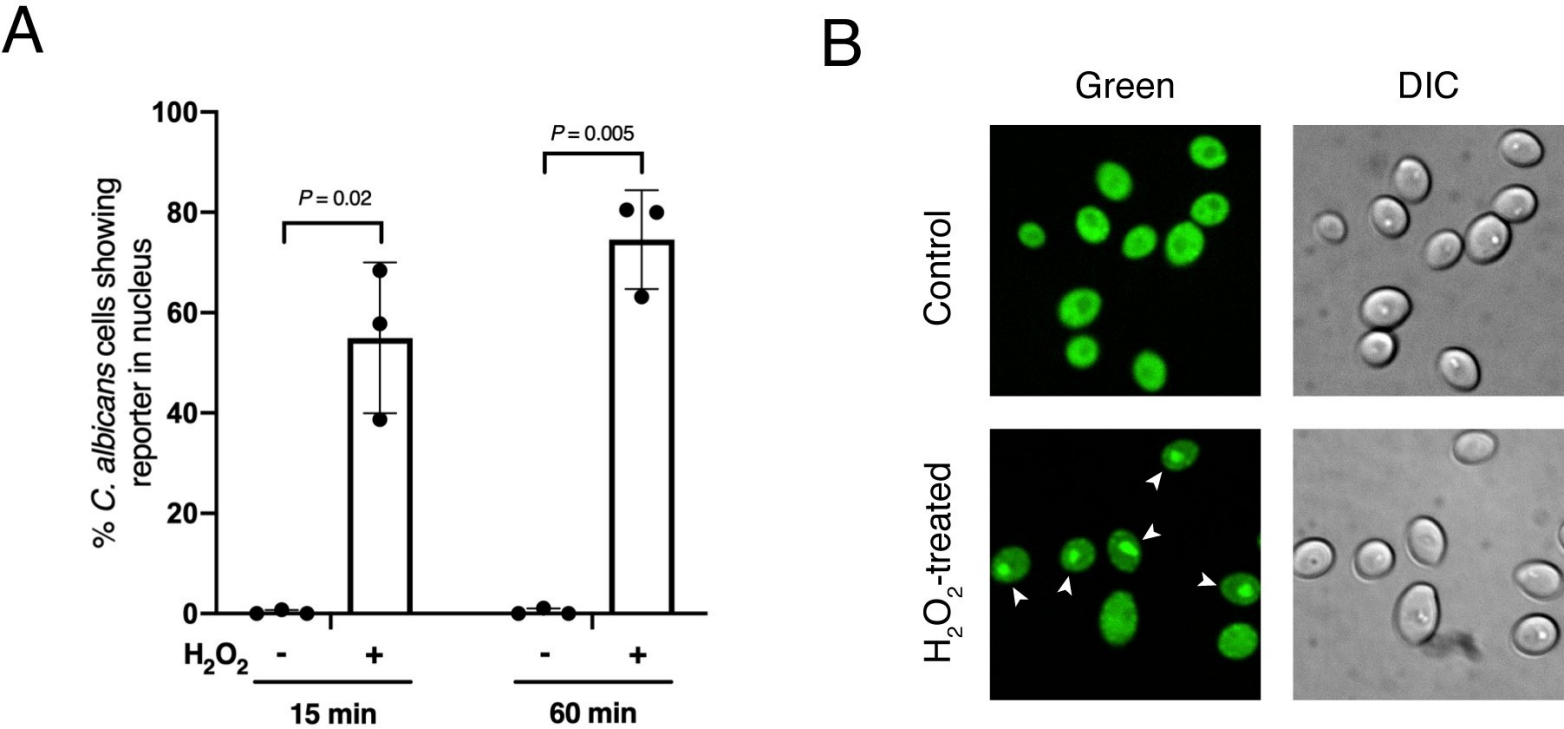
H_2_O_2_ induces Rtg1/3 nuclear localization in *C*. *albicans*. *C*. *albicans* expressing the reporter GFP-Rtg3 was incubated in medium without or with hydrogen peroxide [10 mM]. Shown in (**A**) is the quantification of *C*. *albicans* cells displaying accumulation of the reporter in the nucleus 15 or 60 min after addition of hydrogen peroxide. A minimum of 200 *Candida* cells were scored per time point per experiment. Three independent experiments were performed. Plotted are the means ± SD. Statistical analysis was performed using Student’s *t*-test (two-tailed, two-sample unequal variance). (**B**) Representative images. Arrowheads indicate cells with nuclear accumulation of the reporter.

The results described above strongly suggest that, in the host, one or more ROS can trigger the migration of the Rtg1/3 regulator to the fungal nucleus. If this model were correct, we reasoned that the addition of ROS to the culture medium, independently of the presence of host cells, may be sufficient to promote the accumulation of the regulator in the *Candida* nucleus. One of the intermediate products of the oxidative burst downstream of NOX2 and upstream of MPO is H_2_O_2_. Thus, we quantified the subcellular localization of the GFP-Rtg3 reporter in *Candida* cells incubated in culture medium with or without H_2_O_2_. To avoid secondary effects of ROS, we monitored fluorescence 15 minutes after H_2_O_2_ addition. As shown in Fig 4, and consistent with our prediction, the addition of H_2_O_2_ significantly increased the number of *C*. *albicans* cells displaying accumulation of the regulator in the nucleus. A similar effect was observed 60 minutes after H_2_O_2_ addition (Fig 4) and by exposure to the superoxide generator menadione and the organic peroxide tert-butyl hydroperoxide (S2 Fig). Taken together, the results thus far indicate that ROS is a host signal that *C*. *albicans* Rtg1/3 responds to.

### Expression of *Candida*’s extracellular superoxide dismutase *SOD6* in neutrophils is Rtg1/3-dependent

The finding that ROS can dictate Rtg1/3’s subcellular localization in *C*. *albicans* implied that a role of the regulator may be to activate defenses against oxidative stress. Previous genome-wide chromatin immunoprecipitation and transcriptome analyses uncovered the superoxide dismutases *SOD3* and *SOD6* as targets of Rtg1/3 regulation [9, 11]. While both products are involved in antioxidant defense, *SOD3* encodes a cytoplasmic enzyme that uses manganese as catalytic cofactor [26] whereas *SOD6* encodes an extracellular, copper- and zinc-containing superoxide dismutase [27]. To establish if the expression of either of these two genes is Rtg1/3-dependent in the context of a host ROS environment, we prepared total RNA from the *C*. *albicans* reference strain and an *rtg1 rtg3* double deletion mutant upon engulfment by mouse neutrophils. Because wild-type neutrophils rapidly destroy the *Candida* cells upon engulfment, limiting the recovery of fungal RNA, we used neutrophils derived from MPO^-/-^ mice. As shown in Fig 2, multiple intact *C*. *albicans* cells accumulate inside MPO^-/-^ neutrophils enabling fungal RNA retrieval.

**Fig 5:**
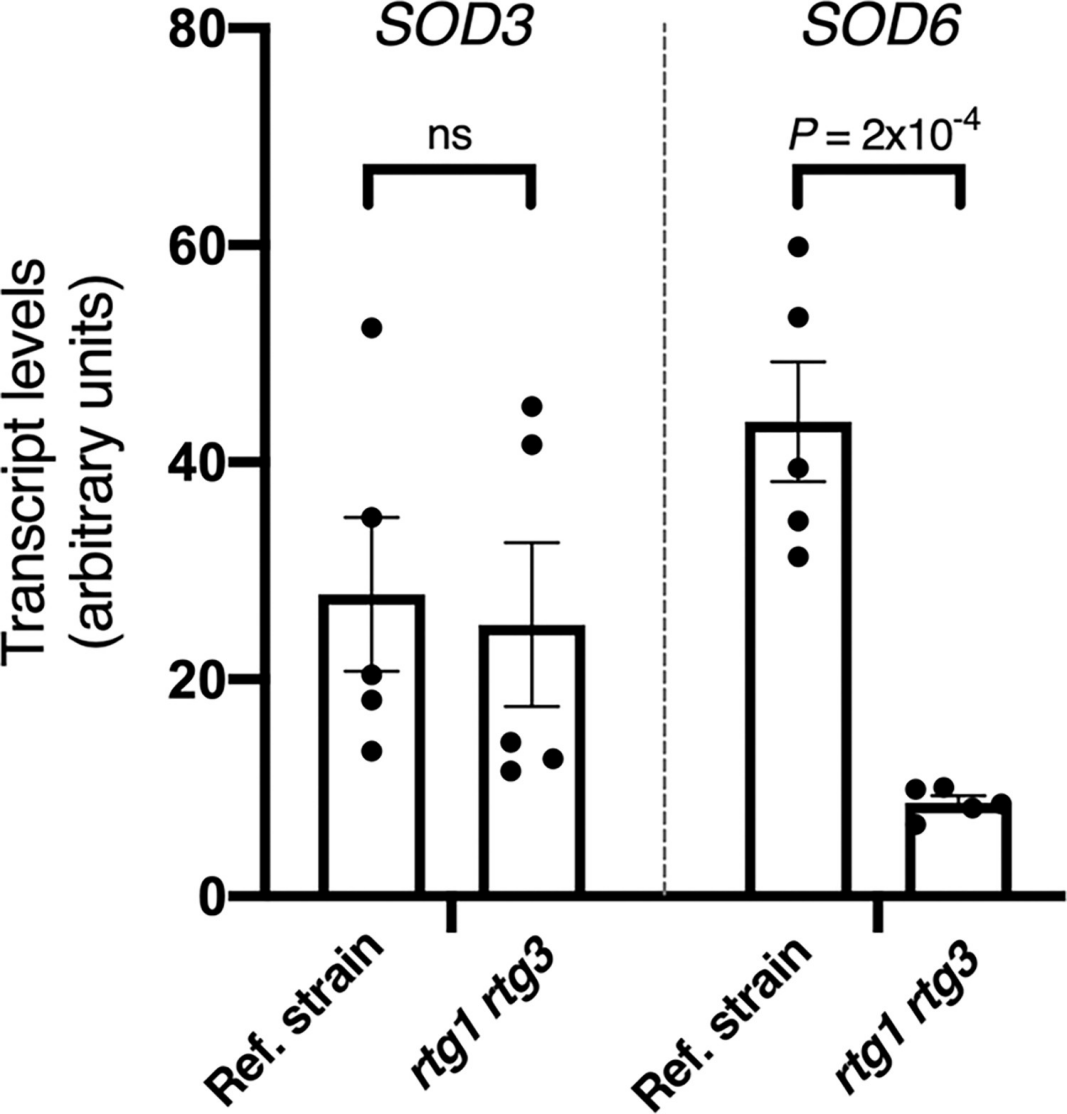
Expression of *C*. *albicans*’ extracellular superoxide dismutase *SOD6* upon uptake by neutrophils is *RTG1*/*3*-dependent. *SOD3* and *SOD6* transcript levels measured by quantitative real-time PCR in *C*. *albicans* reference strain and *rtg1 rtg3* double deletion mutant. Total RNA was prepared from *Candida* cells engulfed by MPO^-/-^ neutrophils. The experimentally validated *TAF10* transcript was used to normalize the qPCR data. Measurements from five biological replicates are included. Plotted are the means ± SEM. Statistical analysis was carried out using Student’s *t*-test (two-tailed, two-sample equal variance).

RT-qPCR analysis of total RNA prepared from *C*. *albicans*-containing neutrophils revealed that that the expression of *SOD6*, but not *SOD3*, was dependent on *RTG1*/*3* (Fig 5). Because Sod6p is an extracellular superoxide dismutase (whereas Sod3p is cytoplasmic), these results are consistent with the notion that Rtg1/3 activates defenses against extracellular ROS rather than cytoplasmic oxidative stress.

### *SOD6* overexpression rescues susceptibility of the *rtg* mutant to killing by neutrophils

**Fig 6:**
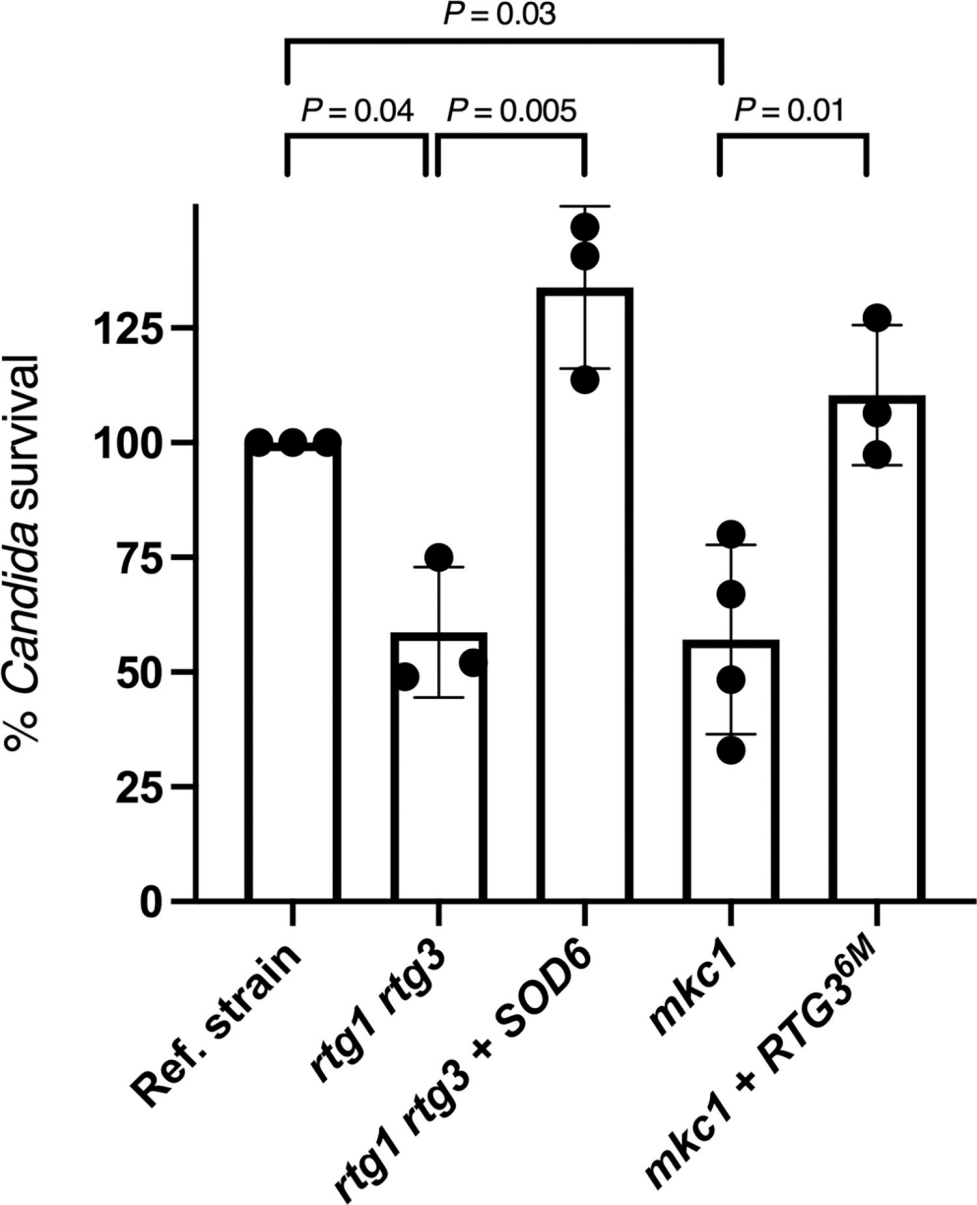
Survival of *C*. *albicans* strains after incubation with mouse neutrophils. Wild-type murine neutrophils were infected with the indicated *C*. *albicans* strains at MOI 0.01 and fungal survival assessed 2 hours post infection. The amount of *C*. *albicans* cells at *t* = 0 was used to calculate survival. Plotted is the percentage survival of each mutant relative to the reference strain. Each dot represents a biological replicate. Means ± SD are indicated. Statistical analysis was carried out using Student’s *t*-test (two-tailed, two-sample equal variance).

We previously established that *rtg3* deletion rendered *C*. *albicans* more susceptible to killing by human neutrophils [11]. Here we show that the *rtg1 rtg3* double mutant strain exhibits a similar phenotype upon incubation with bone marrow derived murine neutrophils (Fig 6). The finding that Rtg1/3 promoted *SOD6* transcription in *C*. *albicans* cells phagocytosed by PMNs (Fig 5) raised the possibility that this superoxide dismutase may be a key determinant of survival inside neutrophils upon Rtg1/3 activation. Consistent with this idea, *SOD6* overexpression rescued the susceptibility of the *rtg1 rtg3* double mutant strain to killing by murine PMNs (Fig 6).

### Identification of *C*. *albicans* kinases implicated in the fungal response to extracellular ROS

The migration of the Rtg1/3 proteins to the nucleus is phosphorylation dependent [11]. We reasoned, then, that there may be ROS-responsive signaling pathway(s) in *C*. *albicans* that determine the phosphorylation status of Rtg1/3. To identify such pathways, we turned to a collection of *C*. *albicans* kinase deletion mutants [28] in which every non-essential protein kinase in the organism has been deleted. The entire collection (86 kinase deletion mutants; two independent isolates for each deleted gene) was probed in two assays: First, sensitivity to hydrogen peroxide and, second, sensitivity to menadione, a drug that generates ROS through redox cycling. The results of both screens are tabulated in S1 Table. Previous studies have shown that *C*. *albicans* mutants that display a phenotype in the presence of hydrogen peroxide usually do so also in the presence of menadione, and *vice versa* [29], which is consistent with our observations (S1 Table). Our two-step screening strategy, therefore, was designed to minimize spurious hits.

Our screen revealed 11 kinase deletion mutants (Table 1) that met the following criteria: (i) increased susceptibility or resistance to hydrogen peroxide and menadione; (ii) no major growth defect in rich (YPD) or defined (SD) medium; and (iii) both isolates showed concordant phenotypes. Three of the hits, *SSK2*, *PBS2* and *HOG1*, are well established components of a signal transduction cascade known as the HOG pathway. Hog1p is a mitogen-activated protein kinase (MAPK) and its upstream protein kinases include the mitogen-activated kinase kinase (MAPKK) Pbs2p and the mitogen-activated kinase kinase kinase (MAPKKK) Ssk2p. This signaling pathway is conserved in yeasts and mediates fungal responses to osmotic, oxidative, and heavy metal stress [30, 31]. Of the remaining eight kinases, only one, Mkc1p, has been shown to be activated upon *Candida* exposure to hydrogen peroxide [32]. Thus, most of our hits represent kinases with previously undescribed roles in ROS responses.

**Table 1:** *C*. *albicans* kinases exhibiting susceptibility/resistance to H_2_O_2_ and menadione.

Systematic name	Standard name	H_2_O_2_ (2mM)	Menadione (30μM)	Known or predicted role
*C4_04450C*	*ATG1*	-1	-2	cytoplasm-to-vacuole targeting pathway
*C7_01330C*	*GCN2*	-1	-2	starvation response
*C6_02190C*	*MCK1*	-1	-2	unknown
*CR_00120C*	*MKC1*	-2	-1	cell wall integrity pathway
*C3_03810W*	*RAD53*	-1	-1	DNA damage response
*C2_07210C*	*TPK2*	+1	+1	filamentation, phenotypic switching and mating
*C4_05030C*	*SSK2*	-2	-2	osmotic and oxidative stress responses
*C2_03330C*	*HOG1*	-1	-2	osmotic and oxidative stress responses
*C3_06070C*	*PBS2*	-1	-2	osmotic and oxidative stress responses
*C5_02560C*		-1	-1	unknown
*C7_04110W*	*ENV7*	-1	-1	unknown

The wild-type phenotype has score = 0. Negative values indicate increased sensibility. Positive values indicate increased resistance. Range of scale: -3 to +3 (complete dataset in S1 Table).

### The *MKC1* and *HOG1* pathways mediate the ROS-dependent regulation of Rtg1/3 in *C*. *albicans*

**Fig 7:**
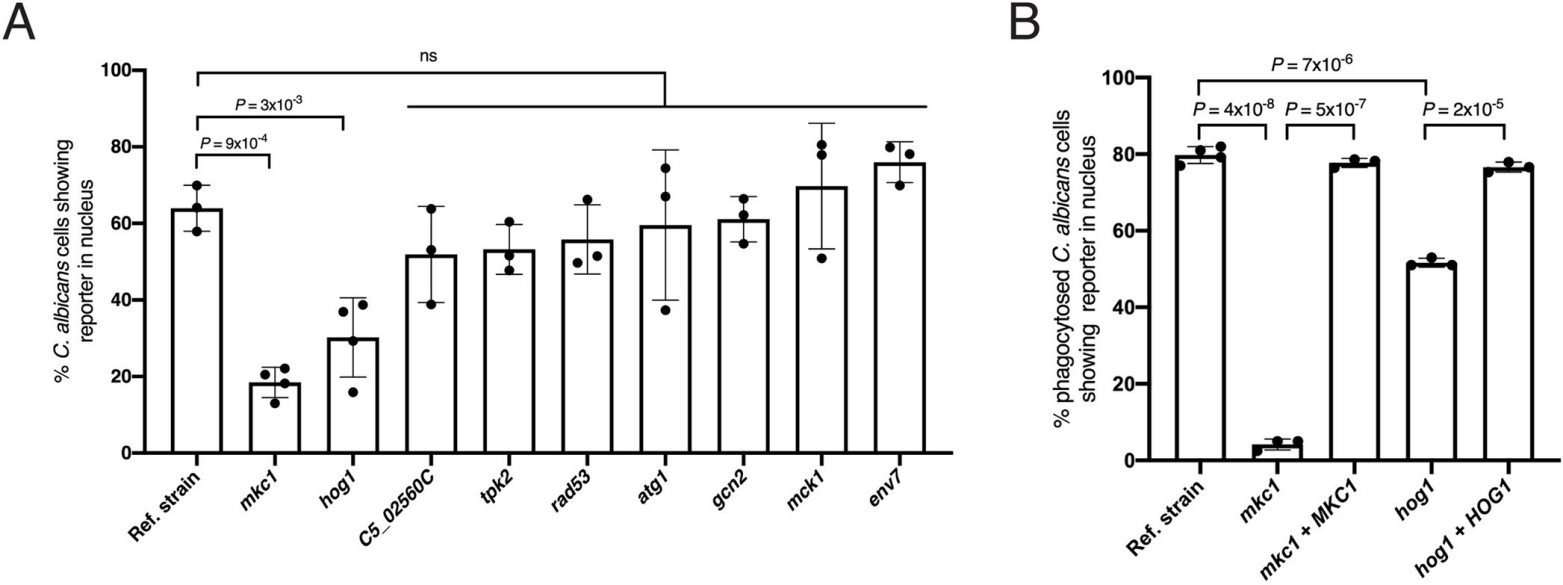
The kinases Mkc1 and Hog1 are necessary for the ROS-dependent nuclear translocation of Rtg1/3. (**A**) *C*. *albicans* kinase deletion mutant strains (from Table 1) expressing GFP- or YFP-Rtg3 reporter were incubated in medium containing hydrogen peroxide. Shown is the quantification of *Candida* cells displaying accumulation of the reporter in the nucleus 15 min after addition of hydrogen peroxide. A minimum of 200 *Candida* cells were scored per strain per experiment. At least three independent experiments were performed. (**B**) Subcellular localization of the Rtg3 reporter in *C*. *albicans* reference strain, *mkc1*, *hog1*, and complemented cells upon uptake by wild-type murine neutrophils. A minimum of 100 *Candida* cells were scored per strain per experiment. At least three independent experiments were carried out. Plotted are the means ± SD. Statistical analysis was conducted using Student’s *t*-test (two-tailed, two-sample unequal variance) with Bonferroni correction.

We next sought to determine whether any of the kinases identified in our screen contributed to establish the subcellular localization of the transcription regulator Rtg1/3. For this, GFP- or YFP-Rtg3p reporters were integrated in each one of nine *C*. *albicans* kinase deletion mutant strains (since *SSK2*, *PBS2* and *HOG1* are shared components of a single pathway, of these 3 kinases only the *hog1* mutant was selected for follow-up). The subcellular localization of the reporter was then evaluated in the presence of hydrogen peroxide. Two deletion mutant strains, *mkc1* and *hog1*, showed a reduction in the reporter’s nuclear localization under these conditions (Fig 7A), suggesting that these two pathways may contribute to the regulator’s ROS-dependent activation.

To probe whether *MKC1* and/or *HOG1* had a role in the activation of Rtg1/3 in the host environment, we evaluated the subcellular localization of the reporter in *C*. *albicans mkc1* and *hog1* mutant cells phagocytosed by neutrophils. As shown in Figs 7B and S3, the *mkc1* mutant, and to a lesser extent the *hog1* mutant, displayed significant reduction in the reporter’s nuclear localization compared to the wild-type reference strain. Adding a wild-type copy of either *MKC1* or *HOG1* to the corresponding deletion strain reverted the phenotypes (Fig 7B). Moreover, the *mkc1* mutation phenocopied, to a large extent, the *rtg1 rtg3* double deletion mutant in fungal survival when challenged *ex vivo* with neutrophils (Fig 6). Furthermore, expression of an *RTG3* allele that encodes a constitutively nuclear regulator [11] restored survival of the *mkc1* mutant upon challenge with neutrophils (Fig 6). Taken together, these findings indicate that *MKC1* is the major kinase implicated in the translocation of the regulator to the *Candida* nucleus upon engulfment by neutrophils (whereas *HOG1* has a relatively minor contribution in this context).

### Rtg1/3 contribute to *C*. *albicans* virulence in an ROS-dependent manner

The results shown thus far indicate that, inside neutrophils, ROS produced by these immune cells determine the subcellular localization of the *C*. *albicans* regulator Rtg1/3. We reasoned that, if the activity of the regulator in *C*. *albicans* indeed responded to host-derived ROS, then Rtg1/3 may play a critical role in defending the fungus against the ROS produced by the host. To test this notion, we turned to an infection model with the nematode *Caenorhabditis elegans*. The worm succumbs to *C*. *albicans* infection in the laboratory and has been shown to be a suitable model to assess the virulence of a variety of *C*. *albicans* strains and the importance of specific genes [33–35]. A key feature of *C*. *elegans* is that it has a single enzyme to produce ROS; nematodes carrying a loss of function mutation in the corresponding gene, *bli-3(im10)*, are viable but defective in ROS production [36] and die more rapidly upon exposure to *C*. *albicans* [37]. Because *RTG1* and *RTG3* are necessary for full *C*. *albicans* virulence in disseminated infections [9], the availability of ROS-deficient worms enabled us to stringently probe whether Rtg1/3’s role during infection was linked to host-derived ROS.

**Fig 8:**
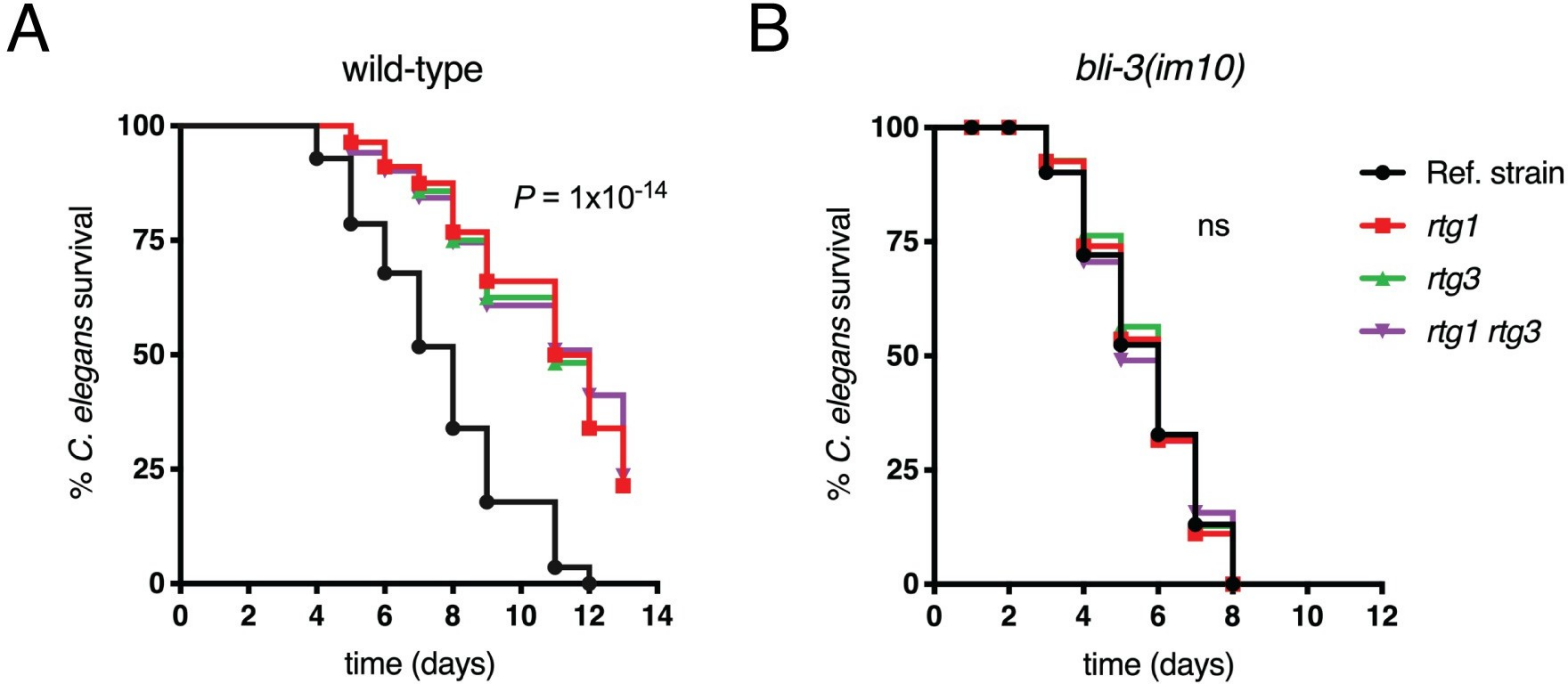
*RTG1*/*3* contributes to *C*. *albicans* virulence in an ROS-dependent manner. Wild-type *C*. *elegans* nematodes (**A**) and *bli-3*(*im10*) mutants impaired in ROS production (**B**) were infected with *C*. *albicans* strains. The three *Candida* deletion strains (*rtg1* and *rtg3* single deletions; and *rtg1 rtg3* double deletion) exhibited reduced virulence compared to the reference strain in wild-type worms (*P* = 1 × 10^−14^) but not in the ROS-deficient *bli-3*(*im10*) worms. The data are representative of experiments repeated three times with an *N* = 60–90 worms for each condition. Statistical analysis was performed using the logrank test (Kaplan-Meier survival curve).

We performed infection assays in wild-type and *bli-3(im10)* nematodes with four different *C*. *albicans* strains: *rtg1* and *rtg3* single mutants, *rtg1 rtg3* double mutant and the wild-type reference strain. The experiments in wild-type worms showed reduced virulence for all three *C*. *albicans* mutants (Fig 8A), in agreement with our previous finding in the standard mouse tail-vein infection model [9]. Adding a wild-type copy of either *RTG1* or *RTG3* to the corresponding single deletion strains reverted the phenotype (S4 Fig). In contrast to the results with wild-type worms, the *C*. *albicans* mutants were indistinguishable from the reference strain in the infection assays with the *bli-3(im10)* worms (Fig 8B). These results indicate that the *RTG1* and *RTG3* genes are dispensable for *C*. *albicans* infection in the absence of host-derived ROS.

## Discussion

**Fig 9:**
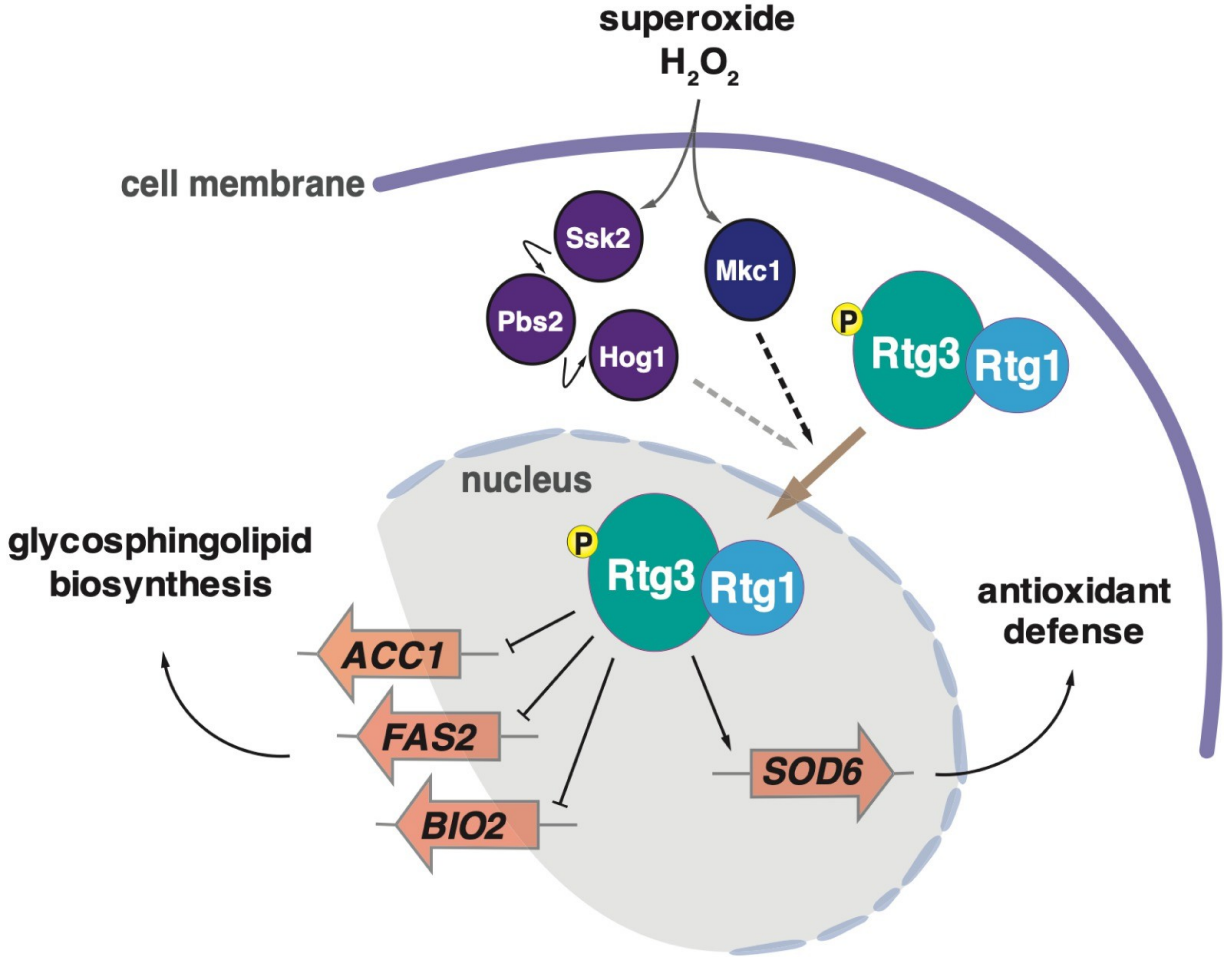
Model depicting the regulation of the Rtg1/3 system in *C*. *albicans*. The Mkc1 kinase, and to a lesser extent the Hog1 signaling cascade, mediate the ROS-dependent translocation to the nucleus of the transcription regulator Rtg1/3. *SOD6*, encoding an extracellular superoxide dismutase, is transcriptionally activated by this heterodimeric transcription factor. *RTG1/3*’s role in sphingolipid biosynthesis is reported elsewhere [11].

The *C*. *albicans* transcription regulator Rtg1/3 is a central component of the fungus’ regulatory network governing mammalian host colonization [9, 22]. The translocation of the regulator to the nucleus, and its concomitant activation, is thought to be triggered by one or more signals present in the fungal cell surroundings. However, the cue(s) inside the host that elicit(s) the nuclear localization of Rtg1/3 have remained unknown. Here we report that host-derived ROS determine the activation of this regulator by promoting its translocation from the cytoplasm to the nucleus of the fungus. Multiple findings support this conclusion: First, the regulator’s migration to the *C*. *albicans* nucleus took place when the fungal cells were inside neutrophils, an immune cell type characterized by producing a robust oxidative burst (Figs 1 and S1). Second, the inhibition of the neutrophil’s main enzyme producing ROS significantly reduced the regulator’s movement to the *Candida* nucleus, even though the ability of the PMNs to engulf the fungus remained unaltered (Figs 2 and 3). Third, the addition of hydrogen peroxide to *Candida* culture medium promoted Rtg1/3 nuclear localization (Fig 4). And fourth, the *C*. *albicans rtg1* and *rtg3* mutants displayed virulence defects in wild-type nematodes but not in worms deficient in ROS production (Fig 8). Taken together, these results indicate that, by responding to host-derived ROS, the *RTG1* and *RTG3* genes enable *C*. *albicans* to withstand oxidative stress inside the host (Fig 9).

How does Rtg1/3 help *C*. *albicans* endure oxidative stress? A previous RNA sequencing study uncovered the superoxide dismutase *SOD3* as the top Rtg1/3-activated gene under laboratory growth conditions [11]. *SOD3* encodes a cytoplasmic superoxide dismutase enzyme that uses manganese as catalytic cofactor and functions in antioxidant defense. Chromatin immunoprecipitation experiments also established that the Rtg1 and Rtg3 proteins bind upstream of the ORF encoding the extracellular, copper- and zinc-containing superoxide dismutase *SOD6* [9]. *SOD3* and *SOD6* are critical components of the *C*. *albicans* response to ROS [27]. Here we establish that the expression of *SOD6* is, at least in part, dependent on *RTG1*/*3* upon engulfment of the fungus by neutrophils (Fig 5). There likely are additional *RTG1*/*3*-regulated products involved in oxidative damage protection; nevertheless, our findings are consistent with the notion that a key function of the regulator is to activate defenses against extracellular ROS.

Previous transcriptome experiments [9, 11] have revealed an eclectic set of transcripts under control of *RTG1*/*3* in *C*. *albicans*. For instance, *RTG1*/*3*-regulated products include genes encoding lipid synthesis enzymes [11], galactose utilization proteins [10], and stress response molecules [9]. Therefore, it is likely that this regulator is used in multiple contexts and in response to diverse stimuli. For instance, Rtg1/3 clearly plays a role in environments where galactose is available, as documented previously [10], and in host environments where ROS accumulate, as shown in this report. Rtg1/3 control of lipid synthesis enzymes [11], which is required for sphingolipid homeostasis, may also be linked to the latter environment because a well-established effect of ROS is to damage biomolecules, including lipids [38, 39]. The regulation of stress response genes, including those encoding protein molecular chaperones [9], may also be rationalized in the context of a response to oxidant damage [40, 41]. Thus, we posit that the response initiated by Rtg1/3 to ROS in the host is designed not only to counteract the ROS molecules themselves (for example by promoting the production of extracellular superoxide dismutases such as *SOD6*) but also to deal with the damage caused by these molecules, particularly on lipids (Fig 9). Alternatively, it also possible that distorted lipid homeostasis/cell wall integrity (as a consequence of ROS-induced damage) is a signal to induce Rtg1/3 activation.

Our search for signaling pathways that may regulate the activity of Rtg1/3 in *C*. *albicans* revealed a primary player, the MAP kinase *MKC1*, and a minor role for *HOG1* (Fig 7). *MKC1* is known for having a major role in the *C*. *albicans* cell wall integrity pathway [42, 43]. Mkc1p is activated by phosphorylation upon membrane perturbation, cell wall stress, and contact with semisolid medium [44]. In addition to being a component of the signal transduction pathway that responds to cell wall stress, the Mkc1p kinase is required for invasive hyphal growth and normal biofilm development [44] as well as virulence in mice [45]. Mkc1p is phosphorylated in the presence of oxidative stress [32] but its downstream targets in the context of the response to ROS have not been defined. Mkc1p’s ortholog in *S*. *cerevisiae*, termed Slt2p or Mpk1p, directs the shuttling between nucleus and cytoplasm of the transcription regulator Swi6p through a mechanism that involves direct phosphorylation [46]. While we provide genetic evidence that links *MKC1* and *RTG1*/*3* in *C*. *albicans*, future work should address whether the latter is a direct target of Mkc1p phosphorylation or if the regulation is indirect. The identification of the Mkc1 kinase as an upstream regulator of the *C*. *albicans* Rtg1/3 system is an important step forward because this is the first report pointing to the existence of a signaling pathway upstream of Rtg1/3 in this fungus. Rtg2, the canonical regulator of Rtg1/3 activity in the model yeast *S*. *cerevisiae* [47], has no ortholog in *C*. *albicans*. Our finding, therefore, paves the way for future studies to address the signaling events that dictate the activation of the *C*. *albicans* Rtg1/3 system.

The involvement of *HOG1* in the ROS-mediated activation of Rtg1/3 was expected because the Hog1 pathway is the most prominent signaling cascade that responds to oxidative stress in *C*. *albicans* and other fungi [30, 31]. It is surprising, however, that the *hog1* mutation has a relatively minor effect on the subcellular localization of Rtg1/3. The Hog1p kinase can phosphorylate the Rtg1 and Rtg3 proteins in *S*. *cerevisiae* [48], suggesting that the link between *HOG1* and Rtg1/3 in *C*. *albicans* may represent a *bona fide* interaction highly relevant under certain conditions; yet in the context evaluated in this report it had limited impact. Furthermore, while usually thought of as two independent signaling pathways, yet-to-be-characterized genetic interactions between *MKC1* and *HOG1* may also be at play because Mkc1p phosphorylation has been found to be, in part, *HOG1*-dependent under some conditions [32].

In addition to the Rtg1/3 system reported here, other fungal transcription factors are known to be regulated by ROS. The *S*. *cerevisiae* Yap1 protein and its ortholog Cap1p in *C*. *albicans* arguably are the best studied examples [49, 50]. These proteins sense ROS through cysteine residues in their C-terminal cysteine-rich domain, form disulfide bonds, accumulate in the nucleus, and regulate transcription of oxidative stress response genes [51, 52]. In *Aspergillus fumigatus* and *A*. *nidulans*, the transcription factor AtfAp responds to oxidative stress by activating genes involved in antioxidant defense mechanisms, including catalases and superoxide dismutases [53]. The *MPKA*- and *SAKA*-mediated stress signaling pathways, as well as the transcription factors Yap1p and Skn7p, also regulate the oxidative stress response in *A*. *fumigatus* [54–57]. Remarkably, single deletion of any of these genes results in no virulence defect implying that there is a significant amount of redundancy in the *A*. *fumigatus* oxidative stress defense system [58]. In *Cryptococcus neoformans*, the transcription factor Skn7p governs the oxidative stress response [59]. ROS stimulate Skn7p phosphorylation and promote nuclear localization of the transcription factor, ultimately resulting in transcriptional activation of antioxidant genes. Skn7 is crucial for *C*. *neoformans* virulence as well [59]. The fact that the regulation of ROS responses and virulence are often integrated underscores the close connection between these two traits in human fungal pathogens.

The nematode *C*. *elegans* allowed us to probe the basis of *RTG1/3*’s contribution to *Candida* virulence. The reduced virulence exhibited by the *rtg1* and *rtg3* mutants in nematodes (Fig 8A) recapitulates the virulence defects observed in the mouse tail-vein infection model [9]. Remarkably, in nematodes lacking ROS production, the *rtg1* and *rtg3* mutants (as well as the *rtg1 rtg3* double mutant) displayed virulence comparable to the *C*. *albicans* reference strain (Fig 8B) indicating that the regulator becomes dispensable for pathogenesis in the absence of host ROS. ROS production is one of the early responses of host innate immunity, rapidly increasing their concentration during infection. In this context, ROS serve to facilitate pathogen clearance although they also contribute to signaling cascades related to inflammation and other immune responses. Our findings support the notion that a major role of *RTG1*/*3* during infection is to orchestrate *C*. *albicans* response to oxidants produced by the host. However, as an input that dictates the activity of Rtg1/3, ROS may concomitantly direct the activation of additional pathways required for *Candida* to endure in the host because Rtg1/3 constitutes a central node of a rather large regulatory network driving mammalian host colonization [22].

In conclusion, our study establishes, for the first time, that reactive oxygen species (ROS) in the host regulate the activity of the *Candida* Rtg1/3 system. Because resolving complex host environments is experimentally challenging, there is a paucity of studies that single out the actual signal(s) that turn on or off microbial regulatory pathways inside the host. In this context, our study constitutes a significant contribution to the field by establishing NOX2-derived ROS as a signal that the fungal Rtg1/3 system responds to.

## Materials and methods

### Ethics statement

Vertebrate animal experiments were conducted in strict accordance with the recommendations in the Guide for the Care and Use of Laboratory Animals as defined by the National Institutes of Health (PHS Assurance #A3413-01). The protocol to retrieve neutrophils from mice was reviewed and approved by the Animal Welfare Committee of The University of Texas Health Science Center at Houston (protocol number AWC-21-0027). Animals were housed at 22°C, ambient humidity, and with a 12 h light-dark cycle.

### Strains and media

All *C*. *albicans* strains used in this study are listed in S2 Table and are derivatives of the clinical isolate SC5314 [60]. The collection of kinase deletion mutants has been described [28] as well as the construction of the *GFP*-*RTG3* and *YFP-RTG3* reporters [9, 11]. The *hog1* deletion and the *rtg1 rtg3* double mutant were constructed as described [61] using the *C*. *albicans* LEUpOUT CRISPR system. The integration of the *RTG3* reporter in selected strains of the *C*. *albicans* kinase deletion library was done by transforming the strains with a PCR product generated with oligos JCP_4368 and JCP_4369, and gDNA from strain JCP_176 or JCP_435 as template. The *SOD6* overexpression strain was constructed by placing the *TDH3* promoter immediately upstream the *SOD6* ORF as described [9]. To generate the *mkc1 RTG3*^6M^ strain, we first constructed an *mkc1 rtg3* double deletion mutant and then transformed this strain with a linearized pSFS2a-derivative plasmid carrying the *YFP*-*RTG3*^6M^ allele [11]. The *hog1* and *mkc1* complemented strains were constructed as reported [28]. Transformants were selected on YPD agar plates containing nourseothricin. The *C*. *albicans* strains were routinely propagated in YPD medium (1% yeast extract, 2% peptone, 2% dextrose). Oligos are listed in S3 Table.

### Neutrophil preparation and *Candida* infection

Human neutrophils were freshly isolated from blood withdrawn from healthy volunteers using the MACSxpress Whole Blood Neutrophil Isolation Kit, Human (Miltenyi Biotec, Cat. No. 130-104-434) according to the manufacturer’s protocol. Mouse neutrophils were isolated from bone marrow freshly harvested from femurs of 8-week-old WT C57BL/6J, MPO^-/-^ (B6.129X1-*Mpo*^*tm1Lus*^/J), or gp91^phox-^ (B6.129S-*Cybb*^*tm1Din*^/J) mice using Histopaque-based density gradient centrifugation as described [62]. 5 × 10^5^ neutrophils were seeded in each well of an 8-well μ-Slide ibiTreat chamber (Ibidi, Cat. No. 80826) containing 150μL of RPMI 1640 medium supplemented with 2% human serum, with and without 30μM diphenyleneiodonium chloride (DPI), and incubated at 37°C and 5% CO_2_ for 20 minutes. Neutrophils were activated by adding 150μL of medium containing 100nM Phorbol Myristate Acetate (PMA) and incubated for 30 minutes at 37°C and 5% CO_2_. Subsequently, the medium was replaced with fresh RPMI 1640 medium (without DPI or PMA) and incubated for 20 minutes at 37°C and 5% CO_2_. Overnight cultures of *C*. *albicans* in YPD were washed twice with PBS, counted in a hemocytometer, and diluted to a concentration of 5 × 10^7^ cells/ml. 10μL of the *C*. *albicans* suspension were added to each well of the neutrophil-containing μ-Slide chamber (MOI = 1) and mixed by gentle pipetting. The chamber slides were briefly centrifuged and placed immediately inside a temperature-controlled chamber (37°C and 5% CO_2_) attached to a fluorescence microscope for imaging.

### Fluorescence microscopy

Live-cell imaging of fungal cells and neutrophils at high resolution was performed in an Olympus IX83 inverted spinning disk confocal fluorescent microscope. The fluorescence intensity and exposure were set at 95% and 1s, respectively. Imaging was carried out in a temperature-controlled chamber attached to the microscope (conditions inside the chamber were set at 37°C and 5% CO_2_). Excitation at 488 nm was used to visualize the subcellular localization of the GFP or YFP reporters. Multiple images of phagocytosed and non-phagocytosed *C*. *albicans* were taken from each μ-Slide’s well at multiple time points. Images were processed using Olympus’ cellSens software and Adobe Photoshop.

### Quantification of subcellular localization

Imaging a large number of cells for quantification (*i*.*e*. to establish the percentage of *Candida* cells displaying nuclear *vs*. cytoplasmic fluorescent signal) was cumbersome under the conditions described above due to the nonsynchronous phagocytosis and killing of the fungus by neutrophils. To circumvent this limitation, after the addition of *C*. *albicans* to neutrophils, the chamber slides were incubated at 37°C and 5% CO_2_ for 20 minutes and subsequently placed on ice for 10 minutes to halt the phagocytosis and killing process. Live-cell imaging was then performed using the same fluorescence microscope described above but at room temperature and without 5% CO_2_. Multiple field images were randomly taken for each well (at 100× magnification) to ensure the scoring of a minimum of 100 engulfed *Candida* cells per well. The experiment was repeated four times. To control for potential confounding effects of time-to-imaging, the DPI-treated samples were imaged first in two replicates whereas the untreated samples were first imaged in the other two repeats.

### Neutrophil ROS production

ROS production by wild-type C57BL/6J mouse neutrophils was quantified using the Cellular ROS Assay Kit (Red) (Abcam, Cat. No. ab186027) following the manufacturer’s protocol. Mouse neutrophils were isolated from bone marrow freshly harvested from femurs of 8-week-old female C57BL/6J mice using Histopaque-based density gradient centrifugation as described [62]. 4 × 10^4^ neutrophils in 50μL of RPMI 1640 were incubated in each well of a 96-well plate at 37°C and 5% CO_2_ for 20 minutes. RPMI medium containing DPI at various concentrations ranging from 2 × 10^−4^ to 2 × 10^2^ μM were prepared. 50 μL of the various concentrations of DPI-RPMI 1640 solutions were added to the wells. Red dye was added to the wells and incubated for 1 hour at 37°C and 5% CO_2_. Neutrophils were subsequently stimulated by adding 20 μL of 11× (550 nM) PMA in PBS to each well and incubating for 30 minutes at 37°C and 5% CO_2_. Red fluorescence was measured using a fluorescence microplate reader at Ex/Em = 520/605 nm (cut-off 590 nm) with bottom read mode. Percentage of ROS production was calculated relative to controls that did not contain DPI. Each DPI concentration was evaluated in triplicates.

### Neutrophil killing assay

The assay was conducted as described [63] using wild-type C57BL/6J mouse neutrophils isolated from the bone marrow.

### H_2_O_2_ assay

The *C*. *albicans* reporter strain was incubated in RPMI 1640 medium with various concentrations of H_2_O_2_ at room temperature. Live imaging was performed using a confocal microscope. Images were taken 15 or 60 minutes after the addition of H_2_O_2_. The subcellular localization of the fluorescent signal was scored in these images. A minimum of 200 *C*. *albicans* cells were scored at each time point in each experiment. Three biological replicates were evaluated.

### RNA isolation and RT-qPCR

*C*. *albicans* cells were incubated with MPO^-/-^ mouse neutrophils in 8-well μ-Slide ibiTreat chambers as described above. After 90 min incubation, the supernatant was removed, and the remaining cells (neutrophils and ingested *Candida*) were scraped off the wells in lysis buffer of the RiboPure RNA purification kit for yeast (Ambion, ThermoFisher Scientific). Total RNA was extracted following the manufacturer’s instructions and concentrated using the RNA Clean & Concentrator-5 kit (ZymoResearch). cDNA was synthesized using SuperScript II Reverse Transcriptase (ThermoFisher Scientific) following the manufacturer’s instructions. Transcript quantification was conducted through real-time PCR analysis using SYBR green. The oligos used are listed in S3 Table. The experimentally validated *TAF10* transcript [64] was used to normalize the qPCR data as we have done before [63].

### Kinase screening

*C*. *albicans* overnight YPD cultures (wild-type parental strain and deletion mutants) were adjusted to an OD_600_ of 2.0. Serial 10-fold dilutions were spotted on synthetic dextrose (SD) agar plates supplemented with H_2_O_2_ (2 mM) or Menadione (30 μM). Scoring was done on images taken after 96 h of incubation at 30°C.

### Caenorhabditis elegans survival assays

The *C*. *elegans* survival assays were carried out using Bristol wild type N2 nematodes as described in previous work with some modifications [35, 37, 65]. To synchronize the nematodes to the same growth stage, L1 worms on non-starved plates were washed off and filtered through a 10μm filter (pluriSelect, pluriStrainer), harvested by centrifugation at 1,500 rpm for 60 seconds, transferred to *cdc-25*.*1* RNAi, rendering them sterile, and grown to the L4 stage.

To prepare the infection plates, fungal strains were grown in YPD broth overnight at 30°C with agitation. 500μl of the culture was plated onto YPD solid medium containing gentamycin (10μg/ml) and grown for 24 hours at 30°C. The synchronized L4 nematodes were then washed off the RNAi plates in 2ml sterile M9 buffer and washed once, at 1,500 rpm for 30 seconds. The animals were infected on *C*. *albicans* lawn for 4 hours at 25°C. Following this exposure, they were washed 4 times with 2 ml of sterile M9 by centrifugation at 750 rpm between washes. The nematodes were then pipetted (~30 per well with two wells per condition for a total of ~60 worms assayed) into six-well plates with 2ml of liquid medium (20% YPD broth and 80% M9). The assays were performed at 25°C and worm survival was scored daily. Kaplan-Meier survival curves were generated and analyzed as described in the statistical analysis section.

### Statistical analyses

Statistical analyses were performed using GraphPad Prism (v. 8.4.3). The quantitative data on the subcellular localization of the reporter and transcript measurements were evaluated using Student’s *t*-test with the parameters described in each figure legend. The Bonferroni correction was used to adjust the *P* value for the multiple comparisons in Fig 7A. Mantel-Cox log rank analysis was used for *C*. *elegans* survival curve comparisons.

## Supporting information

S1 Fig Rtg1/3 translocates to the fungal nucleus upon *Candida* phagocytosis by mouse and human neutrophils. *C*. *albicans* expressing the reporter GFP-Rtg3 was incubated with either (**A**) mouse neutrophils isolated from wild-type C57BL/6J mice or (**B**) neutrophils freshly derived from human blood. At the beginning of the experiment (t = 0), the reporter is distributed throughout the fungal cells. Upon uptake by neutrophils, the reporter accumulates in the *Candida* nuclei (arrowheads).
(TIF)

S2 Fig Exposure to menadione and tert-butyl hydroperoxide promote Rtg1/3 nuclear localization. (**A**) *C*. *albicans* expressing the reporter GFP-Rtg3 was incubated in medium containing tert-butyl hydroperoxide [10 mM] or menadione [100 μM] for ~30 minutes. Shown is the quantification of *C*. *albicans* cells displaying accumulation of the reporter in the nucleus. A minimum of 100 *Candida* cells were scored per time point per experiment. Three independent experiments were performed. Plotted are the means ± SD. Statistical analysis was performed using Student’s *t*-test (two-tailed, two-sample unequal variance). (**B**) Spot assays of both *mkc1* and *hog1* null mutants in presence of H_2_O_2_ [2 mM] or menadione [30 μM].
(TIF)

S3 Fig Subcellular localization of the Rtg3 protein in *mkc1* and *hog1* deletion mutants upon phagocytosis by mouse neutrophils. *C*. *albicans mkc1* and *hog1* single deletion mutants expressing the reporter YFP-Rtg3 were incubated with mouse neutrophils isolated from wild-type C57BL/6J mice and evaluated 15 min after infection. Representative images are shown. Quantification of the subcellular localization is shown in Fig 7B. Engulfed *Candida* cells with reporter in the cytoplasm are indicated with asterisks whereas cells with accumulation of the reporter in the nucleus have arrowheads. The edges of the neutrophils are outlined in the DIC images.
(TIF)

S4 Fig Complementation of the *rtg1* and *rtg3* virulence phenotype in nematodes. Wild-type *C*. *elegans* nematodes were infected with the *C*. *albicans* reference strain, the *rtg1* and *rtg3* single deletions, and their respective gene add-backs. The data are representative of experiments repeated three times with an *N* = 60–90 worms for each condition. Statistical analysis was performed using the logrank test (Kaplan-Meier survival curve).
(TIFF)

S1 Table Oxidative stress screening of *C*. *albicans* kinase deletion collection. (Excel file). (XLSX)

S2 Table List of strains used in this study. (PDF)

S3 Table List of oligos used in this study. (PDF)
