# Supplementary figures and images for: Host-derived reactive oxygen species trigger activation of the Candida albicans transcription regulator Rtg1/3
Source: PLoS Pathog. 2023 Sep 28;19(9):e1011692. doi: 10.1371/journal.ppat.1011692 (PMC10564244; doi:10.1371/journal.ppat.1011692)

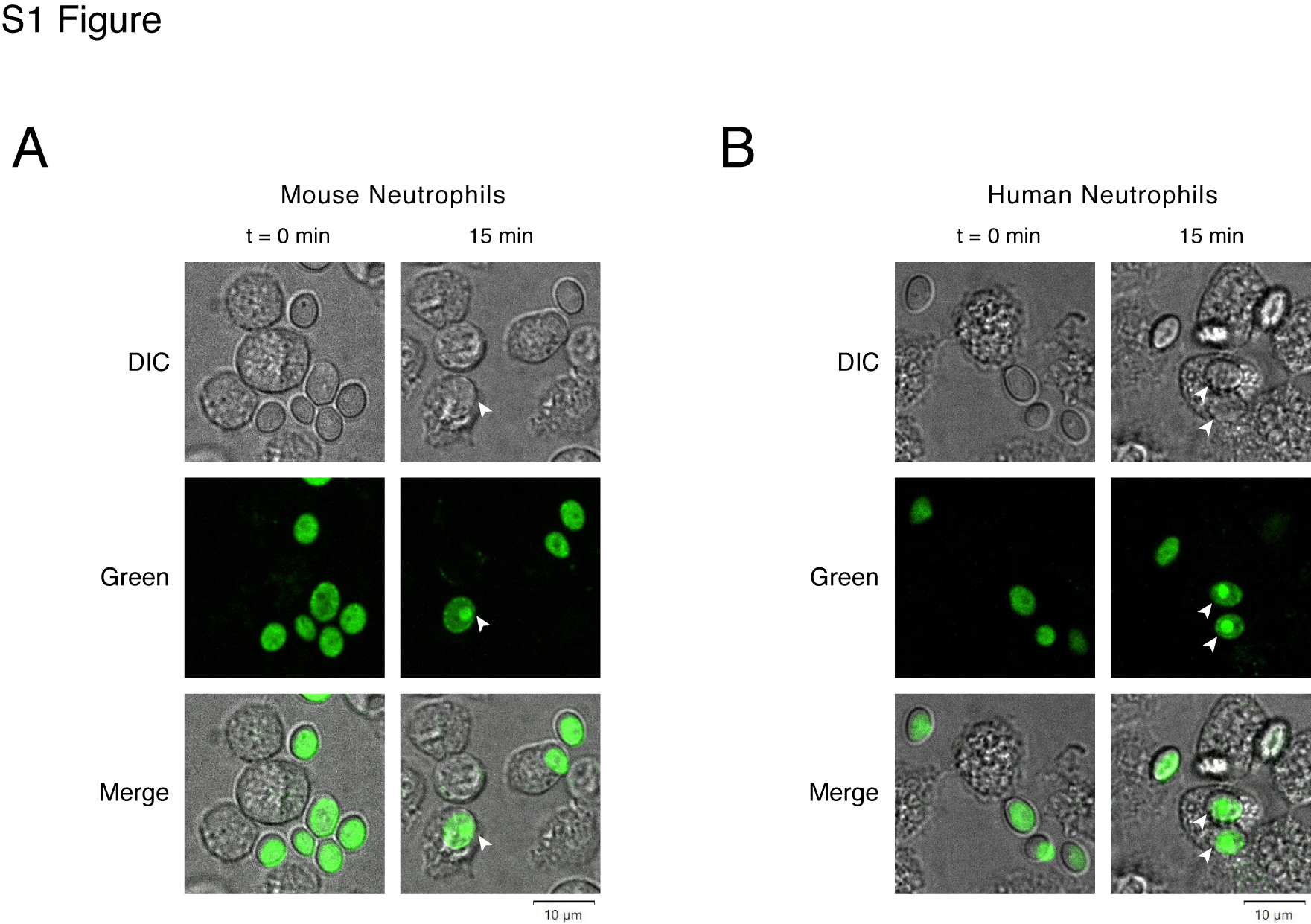

Supplement: S1 Fig — C. albicans expressing the reporter GFP-Rtg3 was incubated with either (A) mouse neutrophils isolated from wild-type C57BL/6J mice or (B) neutrophils freshly derived from human blood. At the beginning of the experiment (t = 0), the reporter is distributed throughout the fungal cells. Upon uptake by neutrophils, the reporter accumulates in the Candida nuclei (arrowheads). (TIF) [file ppat.1011692.s001.tif]

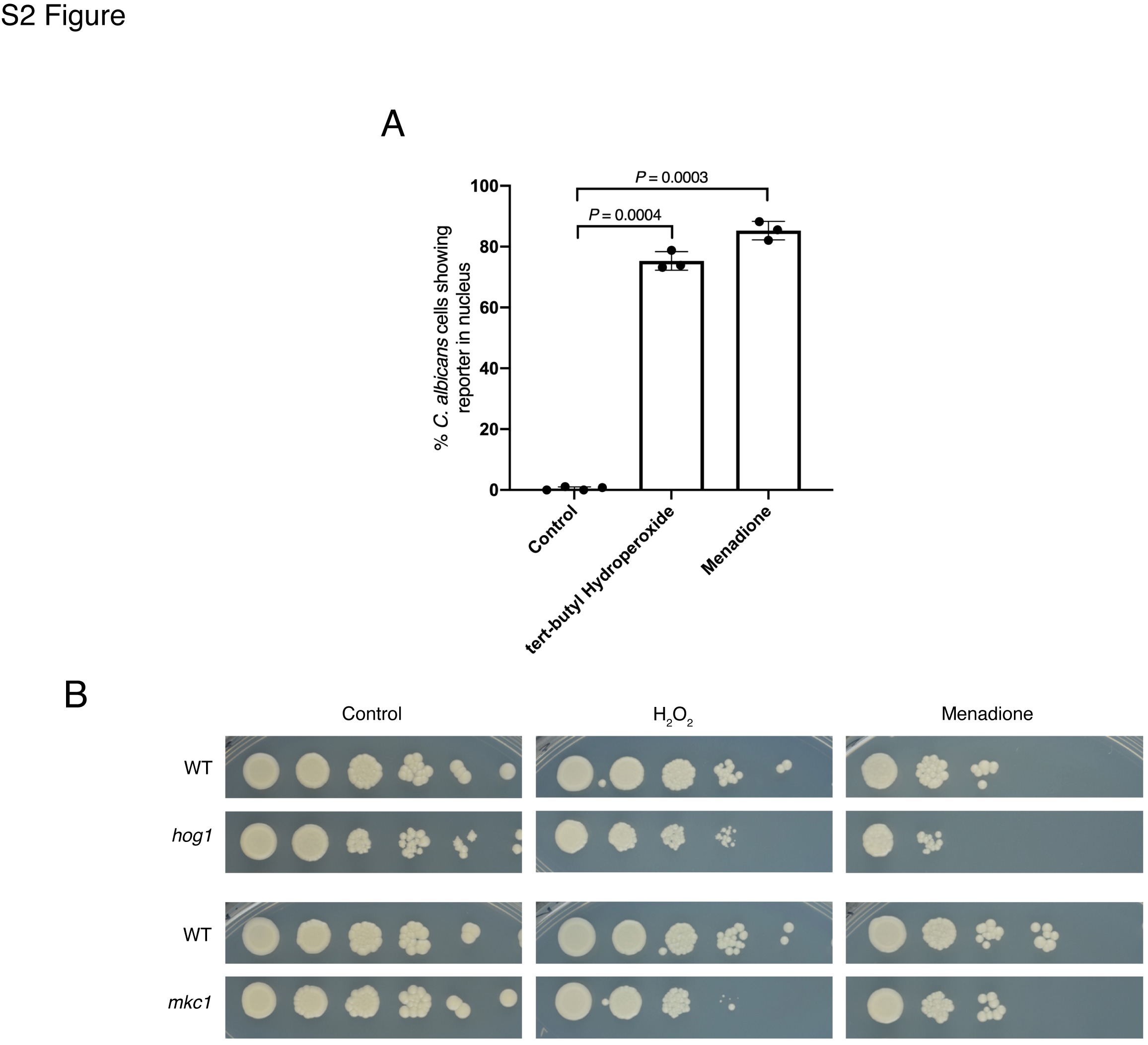

Supplement: S2 Fig — (A) C. albicans expressing the reporter GFP-Rtg3 was incubated in medium containing tert-butyl hydroperoxide [10 mM] or menadione [100 μM] for ~30 minutes. Shown is the quantification of C. albicans cells displaying accumulation of the reporter in the nucleus. A minimum of 100 Candida cells were scored per time point per experiment. Three independent experiments were performed. Plotted are the means ± SD. Statistical analysis was performed using Student’s t-test (two-tailed, two-sample unequal variance). (B) Spot assays of both mkc1 and hog1 null mutants in presence of H2O2 [2 mM] or menadione [30 μM]. (TIF) [file ppat.1011692.s002.tif]

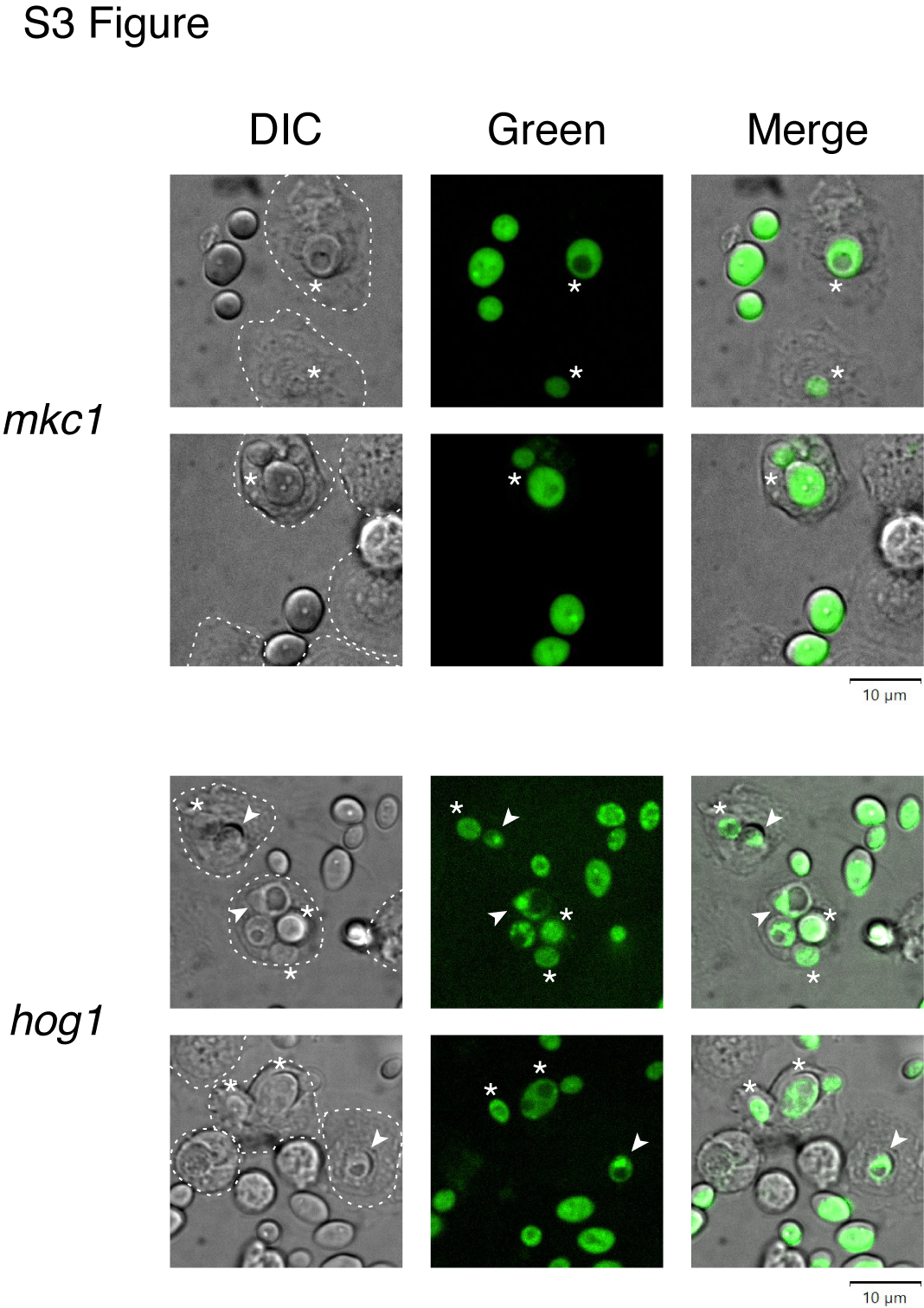

Supplement: S3 Fig — C. albicans mkc1 and hog1 single deletion mutants expressing the reporter YFP-Rtg3 were incubated with mouse neutrophils isolated from wild-type C57BL/6J mice and evaluated 15 min after infection. Representative images are shown. Quantification of the subcellular localization is shown in Fig 7B. Engulfed Candida cells with reporter in the cytoplasm are indicated with asterisks whereas cells with accumulation of the reporter in the nucleus have arrowheads. The edges of the neutrophils are outlined in the DIC images. (TIF) [file ppat.1011692.s003.tif]

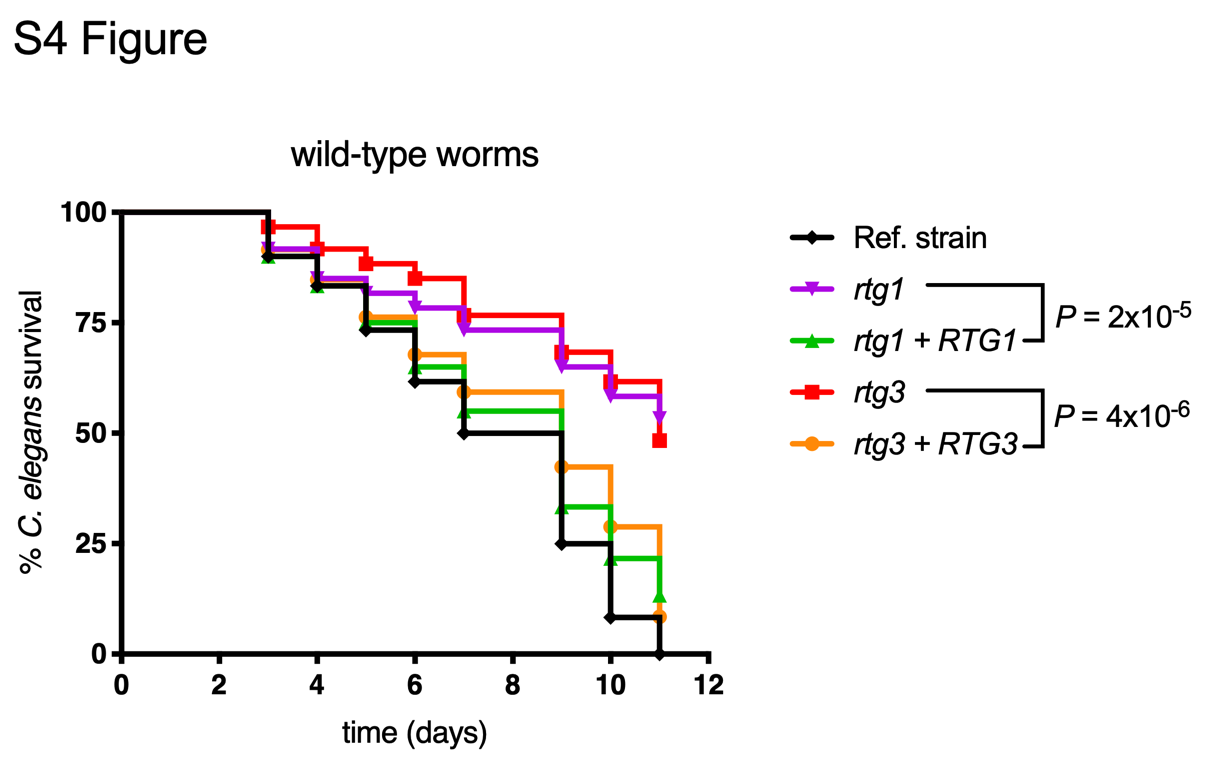

Supplement: S4 Fig — Wild-type C. elegans nematodes were infected with the C. albicans reference strain, the rtg1 and rtg3 single deletions, and their respective gene add-backs. The data are representative of experiments repeated three times with an N = 60–90 worms for each condition. Statistical analysis was performed using the logrank test (Kaplan-Meier survival curve). (TIFF) [file ppat.1011692.s004.tiff]
