# Supplementary material for: Host-derived reactive oxygen species trigger activation of the Candida albicans transcription regulator Rtg1/3
Source: PLoS Pathog. 2023 Sep 28;19(9):e1011692. doi: 10.1371/journal.ppat.1011692 (PMC10564244; doi:10.1371/journal.ppat.1011692)
Supplement: S2 Table — (PDF) [file ppat.1011692.s006.pdf]

S2 Table. Strains used in this study

Strain	Genotype	Source
SC5314	wild-type	[60]
SN152	ura3Δ::λimm434::URA3-IRO1/ura3Δ::λimm434 arg4::hisG/arg4::hisG his1::hisG/his1::hisG leu2::hisG/leu2::hisG	[29]
SN250	ura3Δ::λimm434::URA3-IRO1/ura3Δ::λimm434 arg4::hisG/arg4::hisG his1::hisG/his1::hisG leu2::hisG::CdHIS1/leu2::hisG::CmLEU2	[29]
TF034	ura3Δ::λimm434::URA3-IRO1/ura3Δ::λimm434 arg4::hisG/arg4::hisG his1::hisG/his1::hisG leu2::hisG/leu2::hisG rtg1Δ::CdHIS1/rtg1Δ::CmLEU2	[29]
TF142	ura3Δ::λimm434::URA3-IRO1/ura3Δ::λimm434 arg4::hisG/arg4::hisG his1::hisG/his1::hisG leu2::hisG/leu2::hisG rtg3Δ::CdHIS1/rtg3Δ::CmLEU2	[29]
JCP_191	ura3Δ::λimm434::URA3-IRO1/ura3Δ::λimm434 arg4::hisG/arg4::hisG his1::hisG/his1::hisG leu2::hisG/leu2::hisG rtg3Δ::CdHIS1/rtg3Δ::CmLEU2 rps10Δ::RTG3-SAT1/RPS10	[9]
JCP_194	ura3Δ::λimm434::URA3-IRO1/ura3Δ::λimm434 arg4::hisG/arg4::hisG his1::hisG/his1::hisG leu2::hisG/leu2::hisG rtg3Δ::CdHIS1/rtg3Δ::CmLEU2 rps10Δ::RTG1-SAT1/RPS10	[9]
JCP_175	ura3Δ::λimm434::URA3-IRO1/ura3Δ::λimm434 arg4::hisG/arg4::hisG his1::hisG/his1::hisG leu2::hisG/leu2::hisG RTG3::AgTEF1p-NAT1-AgTEF1UTR-pTDH3-GFP-RTG3/RTG3	[9]
JCP_176	ura3Δ::λimm434::URA3-IRO1/ura3Δ::λimm434 arg4::hisG/arg4::hisG his1::hisG/his1::hisG leu2::hisG/leu2::hisG RTG3::AgTEF1p-NAT1-AgTEF1UTR-pTDH3-YFP-RTG3/RTG3	[9]
JCP_435	ura3Δ::λimm434::URA3-IRO1/ura3Δ::λimm434 arg4::hisG/arg4::hisG his1::hisG/his1::hisG leu2::hisG/leu2::hisG RTG3::AgTEF1p-NAT1-AgTEF1UTR-pTDH3-YFP-RTG3/rtg3Δ::CdHIS1	This study
JCP_1003	ura3Δ::λimm434::URA3-IRO1/ura3Δ::λimm434 arg4::hisG/arg4::hisG his1::hisG/his1::hisG leu2::hisG/leu2::hisG rtg3Δ::CdHIS1/rtg3Δ::CmLEU2 rtg1Δ/rtg1Δ	This study
JCP_1402	ura3Δ::λimm434::URA3-IRO1/ura3Δ::λimm434 arg4::hisG/arg4::hisG his1::hisG/his1::hisG leu2::hisG/leu2::hisG rtg3Δ::CdHIS1/rtg3Δ::CmLEU2 rtg1Δ/rtg1Δ SOD6::AgTEF1p-NAT1-AgTEF1UTR-pTDH3-SOD6/SOD6	This study
AHY940	leu2Δ/LEU2	[61]
JCP_1365	leu2Δ/LEU2 hog1Δ/hog1Δ	This study
JCP_1370	leu2Δ/LEU2 hog1Δ/hog1Δ RTG3::AgTEF1p-NAT1-AgTEF1UTR-pTDH3-YFP-RTG3/RTG3	This study

JCP_1362	RTG3::AgTEF1p-NAT1-AgTEF1UTR-pTDH3-YFP-RTG3 / RTG3	This study
JCP_1354	gcn2Δ RTG3::AgTEF1p-NAT1-AgTEF1UTR-pTDH3-YFP-RTG3 / gcn2Δ RTG3	This study
JCP_1355	mkc1Δ RTG3::AgTEF1p-NAT1-AgTEF1UTR-pTDH3-YFP-RTG3 / mkc1Δ RTG3	This study
JCP_1356	rad53Δ RTG3::AgTEF1p-NAT1-AgTEF1UTR-pTDH3-YFP-RTG3 / rad53Δ RTG3	This study
JCP_1357	orf19.7164Δ RTG3::AgTEF1p-NAT1-AgTEF1UTR-pTDH3-YFP-RTG3 / orf19.7164Δ RTG3	This study
JCP_1359	tpk2Δ RTG3::AgTEF1p-NAT1-AgTEF1UTR-pTDH3-YFP-RTG3 / tpk2Δ RTG3	This study
JCP_1360	orf19.4269Δ RTG3::AgTEF1p-NAT1-AgTEF1UTR-pTDH3-YFP-RTG3 / orf19.4269Δ RTG3	This study
JCP_1363	mck1Δ RTG3::AgTEF1p-NAT1-AgTEF1UTR-pTDH3-YFP-RTG3 / mck1Δ RTG3	This study
JCP_1407	hog1Δ RTG3::AgTEF1p-NAT1-AgTEF1UTR-pTDH3-YFP-RTG3 / hog1Δ RTG3	This study
JCP_1366	atg1Δ RTG3::AgTEF1p-NAT1-AgTEF1UTR-pTDH3-YFP-RTG3 / atg1Δ RTG3	This study
JCP_1399	mkc1Δ pTDH3-YFP-RTG3(6M) / mkc1Δ rtg3Δ	This study
JCP_1408	hog1Δ::HOG1 RTG3::AgTEF1p-NAT1-AgTEF1UTR-pTDH3-YFP-RTG3 / hog1Δ RTG3	This study
JCP_1409	mkc1Δ::MKC1 RTG3::AgTEF1p-NAT1-AgTEF1UTR-pTDH3-YFP-RTG3 / mkc1Δ RTG3	This study
