# Supplementary material for: Host-derived reactive oxygen species trigger activation of the Candida albicans transcription regulator Rtg1/3
Source: PLoS Pathog. 2023 Sep 28;19(9):e1011692. doi: 10.1371/journal.ppat.1011692 (PMC10564244; doi:10.1371/journal.ppat.1011692)
Supplement: S3 Table — (PDF) [file ppat.1011692.s007.pdf]

S3 Table. Oligos used in this study**.**

Name	Description	Sequence (5' to 3')
JCP_4338	gRNA1_CRISPR_HOG1	CGTAAACTATTTTTAATTTGGATGCACGTGATCTTTCCCGGTTTTAGAGCTA GAAATAGC
JCP_4340	dDNA_HOG1_CRISPR_FWD	TAAACAAGTTATAGAAAGAAAATTTTTACAAAGATAAAGCATATAAGAAATC TTCAAAAATACAAGCTAGCAATTATAGAAATAAATTTAAAAGTGAAAT
JCP_4341	dDNA_HOG1_CRISPR_REV	ATTTCACTTTTAAATTTATTTCTATAATTGCTAGCTTGTATTTTTGAAGATT TCTTATATGCTTTATCTTTGTAAAAATTTTCTTTCTATAACTTGTTTA
JCP_4342	C_KO_HOG1_UP_for	AAAACGTTCTCTAGAAATCACATCG
JCP_4343	C_KO_HOG1_DS_rev	TGTTGCTGGAGACGACAAAA
JCP_4344	C_KO_HOG1_ORF_for	CACAAATGACGGGTTACGTG
JCP_4345	C_KO_HOG1_ORF_rev	TCAATAGCCTCCGGTTCAAC
JCP_4368	SAT1_pTDH3_GFP_RTG3_for	GGAAGAAGAGAAAACATCCAC
JCP_4369	SAT1_pTDH3_GFP_RTG3_rev	GCTGGTTTAGCTGAGGTTGC
JCP_4449	SOD6_TDH3_for	GCAGCTTTCATCACAAACAGAACAACAGAACCAAAGTTATTCTTTTACCTAA TCAATACAATCTAAGTTAATCAAGCTTGCCTCGTCCCC
JCP_4450	SOD6_TDH3_rev	TTTTAGGTGACTTGTCTGAAGCAGCTATTGAAACTAGATAGATTAATATGAT AATGGGAATAAAGATCATATTTGAATTCAATTGTGATG
HOG1.01	HOG1_add-back_for	TTTTGAGCTCTCCTTCTTTAACCATGTTTG
HOG1.05	HOG1_add-back_rev	ATACCCGCGGAGAATATTATTAGCAGAAGAC
MKC1.01	MKC1_add-back_for	AAATCGGAGCTCCAAACATTCGTATTAAG
MKC1.05	MKC1_add-back_rev	GAATCCGCGGTTGCAAGATACAAACAC
JCP_4425	SOD3_qPCR_for	GCAAGAGGGATCTCAAGTCG
JCP_4426	SOD3_qPCR_rev	CGGAACTGGTATCAGGGTGT
JCP_4427	SOD6_qPCR_for	TGCTAGTCCAGTTTGCGATG
JCP_4428	SOD6_qPCR_rev	CGACAAATAAGGGTCGCTGT
JCP_4429	TAF10_qPCR_for	GCGGAGACAAAGATCGACAC
JCP_4430	TAF10_qPCR_rev	TATTACTGACGCTGGTGGGG
